# E3 Ubiquitin ligases Cbl-b and c-Cbl maintain the homeostasis of macrophages by regulating the M-CSF/M-CSFR signaling axis

**DOI:** 10.1038/s41419-025-08047-4

**Published:** 2025-10-07

**Authors:** Fei Xu, Chensheng Tan, Kun Tang, Guodong Qiao, Yu Shao, Xiaoping Li, Ji Zhou, Peijie Zhu, Mengyun Wu, Jiamin Cai, Xiu Gao, Yufeng Wang, Beibei Huang, Wenjun Wang, Tian Xia, Xuena Xu, Jiaoyang Li, Zhengrong Chen, Yufang Shi, Chuangli Hao, Yi Yang, Jinping Zhang

**Affiliations:** 1 Institutes of Biology and Medical Sciences, Soochow University, Suzhou, China; 2 Department of Clinical Laboratory, The First Affiliated Hospital of Soochow University, Suzhou, China; 3 Department of Respiratory Medicine, Children’s Hospital of Soochow University, Suzhou, China; 4 Institutes for Translational Medicine, Soochow University, Suzhou, China

**Keywords:** Immune cell death, Immunological disorders

## Abstract

The Casitas B-lineage lymphoma (Cbl) family proteins are E3 ubiquitin ligases implicated in the regulation of various immune cells. However, their function in macrophages remains unclear. Here, we identify both Cbl-b and c-Cbl (Cbls) as inhibitors of macrophage proliferation and promoters of macrophage apoptosis. Mechanically, we identify that Cbls functions upstream of AKT and Erk to mediate the ubiquitination and degradation of M-CSFR. M-CSF stimulation promotes dimerization and autophosphorylation activation of M-CSFR on the macrophage membrane, thereby activating downstream PI3K-AKT and Erk signaling pathways, leading to different biological effects such as macrophage proliferation and survival. At the same time, the Y559 site of the M-CSFR undergoes autophosphorylation, which can promote receptor recruitment and phosphorylation of Cbls. This promotes Cbls to induce K63-linked polyubiquitination at the K791 site of M-CSFR, leading to internalization and degradation of M-CSFR through lysosomal pathways, preventing excessive activation of the signaling pathway. Furthermore, Cbls deficiency results in increased proliferation and decreased apoptosis of macrophages in vitro and in vivo and dKO mice spontaneously develop a macrophage-dominated pulmonary enlargement. Together, these data demonstrate that Cbls play critical roles in the regulation of macrophage homeostasis by inhibiting M-CSFR-mediated AKT and Erk activation.

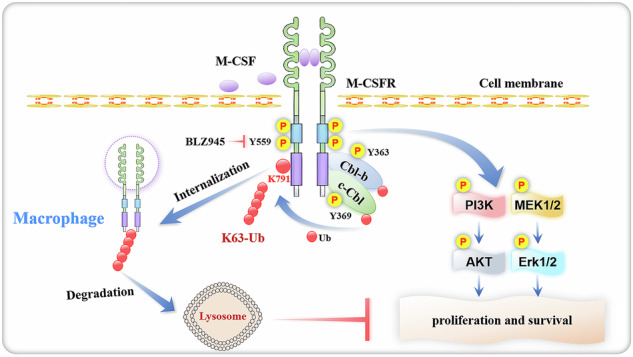

## Introduction

Macrophages are an important part of the immune system, playing key roles in the primary response to pathogens and in coordinating adaptive immune responses, inflammation resolution, tissue homeostasis and repair [1]. In addition, the total number of macrophages has also been shown to be critical for the regulation of the immune system [2–7]. Thus, to ensure the proper function of immune system, the homeostasis, maturation, and total number of macrophages must be tightly regulated. Currently, there remain large gaps in our understanding of the mechanisms underlying the regulation of these processes in macrophages.

Macrophage colony-stimulating factor (M-CSF, also known as CSF-1) is essential for proliferation, survival, and differentiation of macrophages and their precursors [8–10]. M-CSF^op/op^ mice develop osteoporosis due to lack of osteoclasts (OCs) and defects in tissue macrophages and blood monocytes [11]. M-CSF binds to the M-CSF receptor (M-CSFR, also known as CSF-1R or CD115) and activates multiple downstream signaling events [10, 12, 13]. Specifically, the PI3K-AKT and Erk signaling pathways are critical for proliferation and survival of macrophages and their precursors [14–16].

Casitas B-Lymphoma (Cbl) is a family of E3 ubiquitin ligases containing ring finger. In mammals, the Cbl protein family contains of three members: Cbl-b, c-Cbl, and Cbl-3 [17–20], which play key negative regulatory roles in various signaling pathways [21–25]. Systemic double knockout of Cbl-b and c-Cbl (Cbls) resulted in early fetal death in mice, while Cbl-b single knockout or c-Cbl single knockout did not affect birth and lifespan in mice, suggesting that Cbl-b and c-Cbl have important redundant functions in some biological processes. Cbls plays important roles in the prevention of T-cell and B-cell-mediated autoimmune diseases [26, 27]. They regulate T and B cell development, tolerance, and function by regulating signals transmitted by T and B cell antigen receptors and coreceptors [22, 24]. In addition, studies have also found that mice specifically knocked out Cbl-b and c-Cbl in DC cells developed severe spontaneous cirrhosis and liver fibrosis [28, 29]. In contrast to T cells, B cells, and DCs, no studies have yet provided in vivo evidence that Cbls play important roles in macrophage biology, and how Cbls cooperate in vivo macrophages is currently unclear.

Here we found that proliferation of Cbls-deficient macrophages was increased while apoptosis was decreased. Mechanistically, we report that both Cbl-b and c-Cbl are regulators of macrophage proliferation, apoptosis and M-CSF signaling by ubiquitinating and degrading M-CSFR, thereby inhibiting AKT and Erk activation. Studies have demonstrated that binding of M-CSF to M-CSFR activates PI3K and Erk signaling to promote macrophage proliferation and survival [14, 15, 30, 31]. We found that deficiency of Cbls significantly upregulates M-CSFR protein levels, resulting in prolonged activation of AKT and Erk. Moreover Cbls-deficient mice spontaneously develop a macrophage-dominated pulmonary enlargement. Taken together, our data suggest that Cbls exhibit functional redundancy in macrophage homeostasis.

## Results

### Accelerated proliferation and improved survival of Cbls-deficient BMDMs

To investigate whether and how Cbls cooperate in macrophages, we crossed Cbl-b^-/-^ mice with Lyz2-Cre^+^ c-Cbl^flox/flox^ mice to finally obtain four groups of mice: Lyz2-Cre^-^ c-Cbl^flox/flox^ Cbl-b^+/+^ (wild-type or WT, thereafter), Lyz2-Cre^-^ c-Cbl^flox/flox^ Cbl-b^-/-^ (Cbl-b KO, thereafter), Lyz2-Cre^+^ c-Cbl^flox/flox^ Cbl-b^+/+^ (c-Cbl cKO, thereafter) and Lyz2-Cre^+^ c-Cbl^flox/flox^ Cbl-b^-/-^ (dKO, thereafter) mice (Supplementary Fig. 1A). Deletion of c-Cbl mediated by Lyz2-Cre occurs predominantly in myeloid cells (including macrophages), and deletion of Cbls in BMDMs was confirmed at protein level by Western blot (Supplementary Fig. 1B–D). All four types of mice were born at normal Mendelian frequencies (Supplementary Fig. 1F).

**Fig. 1:**
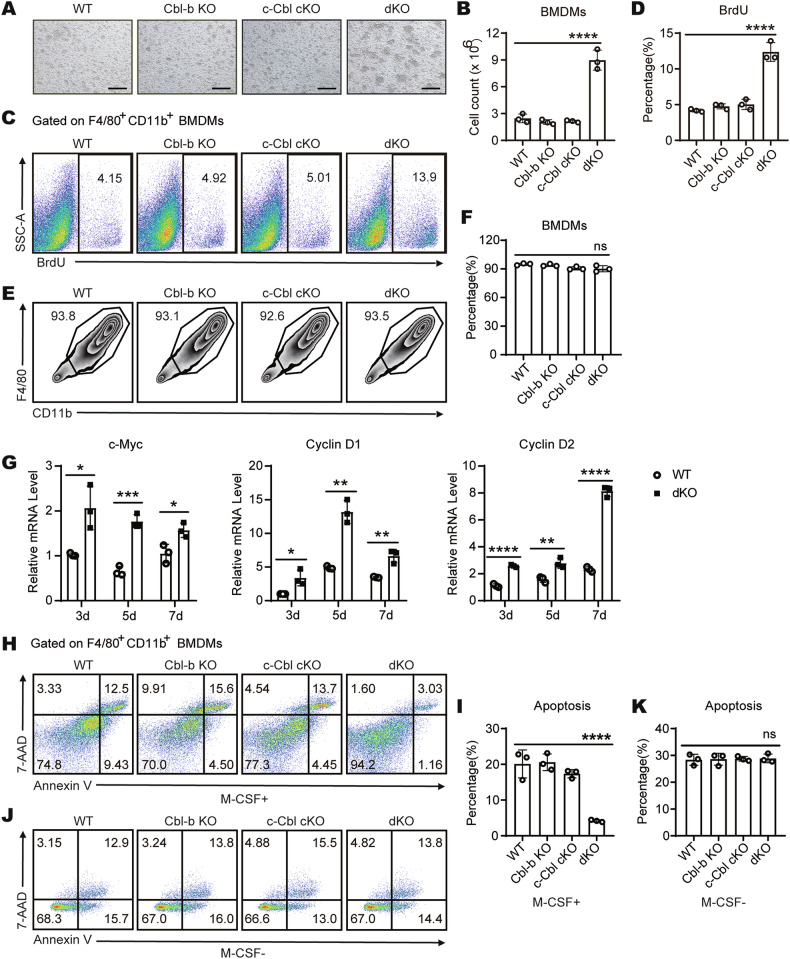
Accelerated proliferation and improved survival of Cbls-deficient BMDMs. **A** Microscopy of clonal morphology of WT, Cbl-b KO, c-Cbl cKO and dKO BMDMs generated in M-CSF dependent BM cell culture (*r* = 3 per group); scale bar, 100 μm. **B** Statistics of absolute number of WT, Cbl-b KO, c-Cbl cKO and dKO BMDMs generated in M-CSF dependent BM cell culture (*r* = 3 per group). Proliferation of WT, Cbl-b KO, c-Cbl cKO and dKO BMDMs generated in M-CSF dependent BM cell culture. Shown are FACS analyses **C** and statistics **D** of BrdU^+^ F4/80^+^ CD11b^+^ cells (*r* = 3 per group). WT, Cbl-b KO, c-Cbl cKO and dKO BMDMs were cultured in the presence of M-CSF (20 ng/mL) for 7 days. Then proportion of macrophages was analyzed by flow cytometry. FACS analyses **E** and statistics **F** of F4/80^+^ CD11b^+^ cells (*r* = 3 per group). **G** Quantitative PCR analysis of the expression levels of cell cycle-related genes *c-Myc*, *cyclin D1* and *cyclin D2* in WT and dKO BMDMs cultured in the presence of M-CSF (20 ng/mL) for the indicated times (*r* = 3 per group). WT, Cbl-b KO, c-Cbl cKO and dKO BMDMs were cultured in the presence of M-CSF (20 ng/mL) for 7 days. Then apoptosis rates were analyzed by flow cytometry. FACS analyses **H** and statistics **I** of Annexin V^+^ 7AAD^−^ and Annexin V^+^ 7AAD^+^ F4/80^+^ CD11b^+^ cells (*r* = 3 per group). WT, Cbl-b KO, c-Cbl cKO and dKO BMDMs were cultured in the presence of M-CSF (20 ng/mL) for 5 days, followed by culturing in M-CSF-free medium for another 2 days. Then apoptosis rates were analyzed by flow cytometry. FACS analyses **J** and statistics **K** of Annexin V^+^ 7AAD^−^ and Annexin V^+^ 7AAD^+^ F4/80^+^ CD11b^+^ cells (*r* = 3 per group). The “*r*” represents the number of times the technology is repeated. One-Way ANOVA comparisons for **B, D, F, I** and **K**, unpaired Student’s *t* test for **G**. *ns*, no significance, *p < 0.05, **p < 0.01, ***p < 0.001, ****p < 0.0001. *p* < 0.05 was considered statistically significant.

To address the potential role of Cbls in macrophage development, we cultured BMDMs from WT, Cbl-b KO, c-Cbl cKO, and dKO mice with M-CSF and observed that the dKO BM cells produced more, larger, and denser macrophage clones under M-CSF stimulation compared to the control groups (Fig. 1A), and the total number of macrophages obtained by differentiation of dKO BM cells with M-CSF also increased significantly (Fig. 1B). These results lead us to further investigate the role of Cbls in macrophage development in response to M-CSF.

The increased number of macrophages might be due to more efficient proliferation and/or less cell death. Under the same initial number of bone marrow (BM) cells, these cells were induced to differentiate into BMDMs using a consistent concentration of M-CSF. We quantified the proliferation of WT, Cbl-b KO, c-Cbl cKO, and dKO BMDMs by analyzing BrdU incorporation. The results showed that the proportion of BrdU^+^ proliferating cells in dKO BMDMs (cells double positive for F4/80 and CD11b) was significantly increased compared to the control groups (Fig. 1C, D). However, when compared to the control groups, the proportion of dKO BMDMs—defined as the percentage of F4/80 and CD11b double-positive cells—did not exhibit any significant change (Fig. 1E, F). These findings suggest that the primary factor contributing to the increased number of dKO BMDMs is an enhanced intrinsic capacity for division and growth (proliferation), rather than an improved transformation efficiency during differentiation (induction ratio). To verify the role of Cbl-b and c-Cbl in cell cycle progression, we measured the expression levels of mRNA transcripts encoding cell cycle-regulatory proteins in WT and dKO BMDMs cultured with M-CSF for 3, 5 and 7 days. dKO BMDMs expressed more c-Myc, Cyclin D1 and Cyclin D2 mRNA at day 3, 5 and 7 compared with WT BMDMs (Fig. 1G). These data suggest that the absence of Cbls promotes M-CSF-induced macrophage proliferation.

We next assessed whether Cbls deficiency influenced cell death. We examined the apoptosis rates of WT, Cbl-b KO, c-Cbl cKO and dKO BMDMs in the presence of M-CSF. The results showed that the percentage of apoptosis of dKO BMDMs decreased significantly compared to the other three groups of cells (Fig. 1H, I). We also evaluated whether the effects of deficiencies of Cbl-b and c-Cbl on macrophage apoptosis were dependent on the presence of M-CSF. WT, Cbl-b KO, c-Cbl cKO and dKO BMDMs were cultured in the presence of M-CSF for 5 days, and then switched to M-CSF-free medium for 2 more days. After M-CSF deprivation, there was no significant change in apoptosis percentage of dKO BMDMs compared with the control groups (Fig. 1J, K). These data suggest that the absence of Cbls contributes to M-CSF-induced macrophage survival. Collectively, our findings demonstrate that Cbls negatively regulate M-CSF-induced macrophage proliferation and promoted macrophage apoptosis.

To establish that the abnormal phenotypes observed in dKO BMDMs were indeed attributable to the deletion of Cbl-b and c-Cbl, rather than resulting from off-target effects or other confounding factors, we conducted “rescue” experiments. We infected bone marrow cells from dKO mice with OE-Ctrl, OE-Cbl-b, OE-c-Cbl, and OE-Cbls viruses while simultaneously inducing cell differentiation into BMDMs using M-CSF. We observed that compared with the OE-Ctrl group, the overexpression of either Cbl-b or c-Cbl alone, as well as their simultaneous overexpression, significantly inhibited the formation of macrophage clones (Supplementary Fig. 2A, B). Meanwhile, we employed flow cytometry to assess cell proliferation and apoptosis. The results showed that compared with the OE-Ctrl group, the overexpression of either Cbl-b or c-Cbl alone, as well as their simultaneous overexpression, significantly inhibited the proliferation of dKO BMDMs (Supplementary Fig. 2C, D) while promoting their apoptosis (Supplementary Fig. 2E, F). These data suggest that the phenotype observed in dKO BMDMs is indeed attributable to the loss of protein function of Cbls.

### dKO BMDMs exhibit prolonged AKT and Erk activation upon M-CSF stimulation

**Fig. 2:**
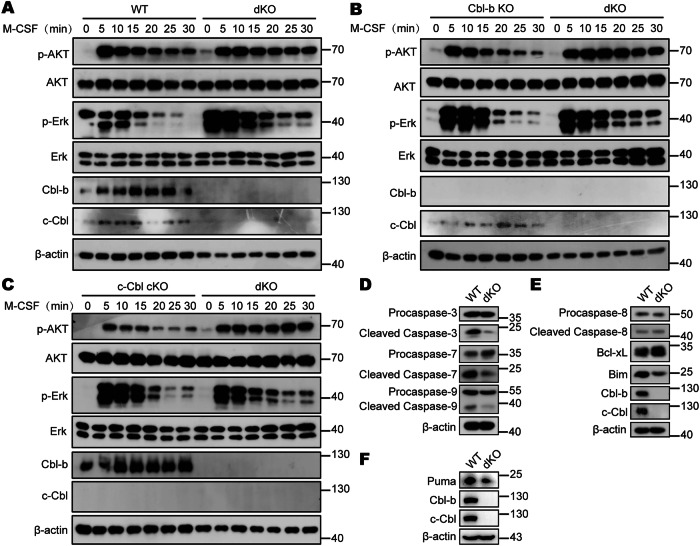
dKO BMDMs exhibit prolonged AKT and Erk activation upon M-CSF stimulation. **A** WT and dKO BMDMs were starved of M-CSF for 24 h and restimulated with M-CSF (50 ng/mL) for indicated times and harvested for western blot analysis of p-AKT and p-Erk. **B** Cbl-b KO and dKO BMDMs were starved of M-CSF for 24 h and restimulated with M-CSF (50 ng/mL) for indicated times and harvested for western blot analysis of p-AKT and p-Erk. **C** c-Cbl cKO and dKO BMDMs were starved of M-CSF for 24 h and restimulated with M-CSF (50 ng/mL) for indicated times and harvested for western blot analysis of p-AKT and p-Erk. **D** Western blot analysis of cleaved caspase-3, -7, -9 in WT and dKO BMDMs generated in M-CSF dependent BM cell culture. **E** Western blot analysis of cleaved caspase-8, Bim and Bcl-xL in WT and dKO BMDMs generated in M-CSF dependent BM cell culture. **F** Western blot analysis of Puma in WT and dKO BMDMs generated in M-CSF dependent BM cell culture.

The excessive proliferation and improved survival of M-CSF-induced dKO macrophages suggests that Cbls are involved in the regulation of M-CSF signaling. Studies have shown that binding of M-CSF to M-CSFR activates PI3K-AKT and Erk signaling to promote macrophage proliferation and survival. We therefore examined whether deficiencies of Cbls enhanced PI3K-AKT and Erk signaling. Thus, we sought to stimulate WT, Cbl-b KO, c-Cbl cKO, and dKO BMDMs with M-CSF for various times and analyzed these signaling pathways. Sustained phosphorylation of AKT, the major target of PI3K, was detected in dKO macrophages (Fig. 2A–C). Although p-Erk levels in WT, Cbl-b KO, c-Cbl cKO, and dKO BMDMs had decreased to lower levels within 20–30 min after M-CSF stimulation, p-Erk expression levels in dKO BMDMs remained higher than that of the control group (Fig. 2A–C). In addition, we observed that high levels of p-AKT were still detectable in dKO BMDMs 6 h after M-CSF stimulation compared to control group (Supplementary Fig. 3A–C). These results indicate that knockout of Cbls significantly prolong the activation of AKT and Erk under M-CSF stimulation. Taken together, these findings suggest that Cbls may be involved in macrophage proliferation by regulating the M-CSF/M-CSFR signaling pathway.

In order to further explore the molecular mechanism of Cbls involved in macrophage apoptosis by regulating M-CSF/M-CSFR signaling pathway. We detected the expression levels of apoptosis-related proteins in WT and dKO BMDMs induced with M-CSF by Western blot. It is well known that the Caspase family plays a crucial role in the process of apoptosis, and Cleaved Caspase-3 and Cleaved Caspase-7 are the key executive molecules [32, 33]. The above flow detection results showed that in the presence of M-CSF, the proportion of apoptotic cells in dKO BMDMs was significantly reduced compared with WT BMDMs (Fig. 1H, I). We further confirmed this result by using Western blot to measure the cleavage of effector caspases downstream of apoptosis pathway, including caspase-3 and caspase-7. Consistently, the cleavage of caspase-3, caspase-7 and caspase-9 were much lower in dKO BMDMs, with no change for intact forms (Fig. 2D). In addition, the protein expression levels of pro-apoptotic molecule Bim in dKO BMDMs were significantly decreased, while the protein expression levels of the anti-apoptotic molecule Bcl-xL were significantly increased (Fig. 2E). Puma (p53 upregulated modulator of apoptosis) is also one of the most well-known apoptotic inducers [34, 35]. Puma activates caspase-9 by antagonizing Bcl-2/Bcl-xL and promoting the release of Bax/Bak [36–38]. Recent study has shown that AKT inactivates Puma at proteasomal level to prevent apoptosis [39]. We also measured the protein expression levels of Puma in WT and dKO BMDMs. The results showed that the protein expression level of Puma in dKO BMDMs was significantly lower than that in WT BMDMs (Fig. 2F). Taken together, these findings suggest that Cbls may be involved in macrophage apoptosis by regulating the M-CSF/M-CSFR signaling pathway.

### Cbls collaboratively inhibit M-CSF-dependent cell proliferation and promote cell apoptosis by down-regulating M-CSFR protein levels

**Fig. 3:**
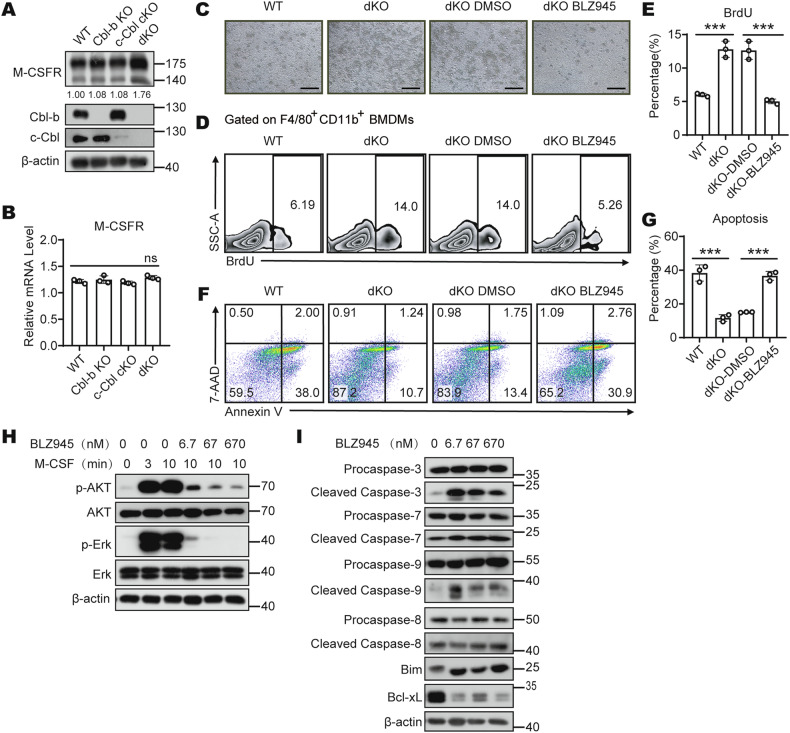
Cbls collaboratively inhibit M-CSF-dependent cell proliferation and promote cell apoptosis by down-regulating M-CSFR protein levels. **A** Western blot analysis of M-CSFR expression in WT, Cbl-b KO, c-Cbl cKO and dKO BMDMs. And the data was quantified using Image J software. The molecular masses of the mature M-CSFR (175 kDa) and its precursor (140 kDa) are indicated. **B** Quantitative PCR analysis of M-CFSR mRNA levels in WT, Cbl-b KO, c-Cbl cKO and DKO BMDMs (*r* = 3 per group). **C** Microscopy of clonal morphology of WT, dKO, dKO (DMSO) and dKO (BLZ945) BMDMs generated in M-CSF dependent BM cell culture (r = 3 per group); scale bar, 100 μm. Proliferation of WT and dKO BMDMs generated in M-CSF dependent BM cell culture which added BLZ945. Shown are FACS analyses **D** and statistics **E** of BrdU^+^ F4/80^+^ CD11b^+^ cells (*r* = 3 per group). Apoptosis of WT and dKO BMDMs generated in M-CSF dependent BM cell culture which added BLZ945. Shown are FACS analyses **F** and statistics **G** of Annexin V^+^ 7AAD^−^ and Annexin V^+^ 7AAD^+^ F4/80^+^ CD11b^+^ cells (*r* = 3 per group). **H** dKO BMDMs were starved of M-CSF for 24 h and treated with M-CSF (50 ng/mL) and different concentrations of BLZ945 for indicated times and harvested for western blot analysis of p-AKT and p-Erk. **I** dKO BMDMs treated with different concentrations of BLZ945 and harvested for western blot analysis of cleaved caspase-3, -7, -8, -9, Bim and Bcl-xL. The “*r*” represents the number of times the technology is repeated. One-Way ANOVA comparisons for **B**, unpaired Student’s *t* test for **E** and **G**. *ns*, no significance, ***p < 0.001. *p* < 0.05 was considered statistically significant.

Since the function of M-CSF is mediated by its specific receptor M-CSFR, we hypothesized that Cbls may modulate M-CSF/M-CSFR signaling by influencing M-CSFR expression. To further confirm the regulatory relationship between Cbls and M-CSF/M-CSFR signaling pathway. We examined M-CSFR expression in WT, Cbl-b KO, c-Cbl cKO, and dKO BMDMs. Western Blot results showed significantly higher M-CSFR protein levels in dKO BMDMs compared to the other three control cells (Fig. 3A). However, qPCR results showed that Cbls knockout did not affect M-CSFR mRNA expression (Fig. 3B). These results suggest that Cbls synergistically down-regulate M-CSFR protein levels.

To verify the positive role of M-CSFR on macrophage proliferation in dKO mice, specific inhibitor BLZ945 for M-CSFR was added into the culture system used for induction of BMDMs, and then cell proliferation was assessed by flow cytometry. The results showed the addition of BLZ945 significantly inhibited the formation of macrophage clones in dKO mice (Fig. 3C) and significantly decreased the percentage of proliferating dKO BMDMs (Fig. 3D, E). To further verify the negative role of M-CSFR on macrophage apoptosis in dKO mice, specific inhibitor BLZ945 for M-CSFR was added into the culture system used for induction of BMDMs, and then cell apoptosis was assessed by flow cytometry. The results showed the addition of BLZ945 increased the percentage of apoptotic dKO BMDMs (Fig. 3F, G).

Next, M-CSF was used to stimulate dKO BMDMs at different time points and BLZ945 was combined to treat the cells. Finally, Western blot was used to analyze the activation of M-CSF/M-CSFR downstream signaling pathway. The results showed that the levels of p-AKT and p-Erk in dKO BMDMs were significantly increased after M-CSF stimulation, whereas the phosphorylation of AKT and Erk induced by M-CSF stimulation was effectively reduced after BLZ945 treatment of dKO BMDMs (Fig. 3H). We also treated dKO BMDMs with BLZ945, and then analyzed the expression levels of apoptosis-related proteins downstream of M-CSF/M-CSFR signaling pathway by Western blot. The results showed that the treatment of dKO BMDMs with BLZ945 resulted in protein expression levels of Cleaved caspase-3, Cleaved caspase-7, Cleaved caspase-9 and the pro-apoptotic molecule Bim were significantly increased, while protein expression levels of the anti-apoptotic molecule Bcl-xL were significantly decreased (Fig. 3I). Taken together, these findings suggest that M-CSFR indeed participated the proliferation and apoptosis of macrophages controlled by Cbls.

### Cbls mediate the ubiquitination and subsequent degradation of M-CSFR in response to M-CSF induction

**Fig. 4:**
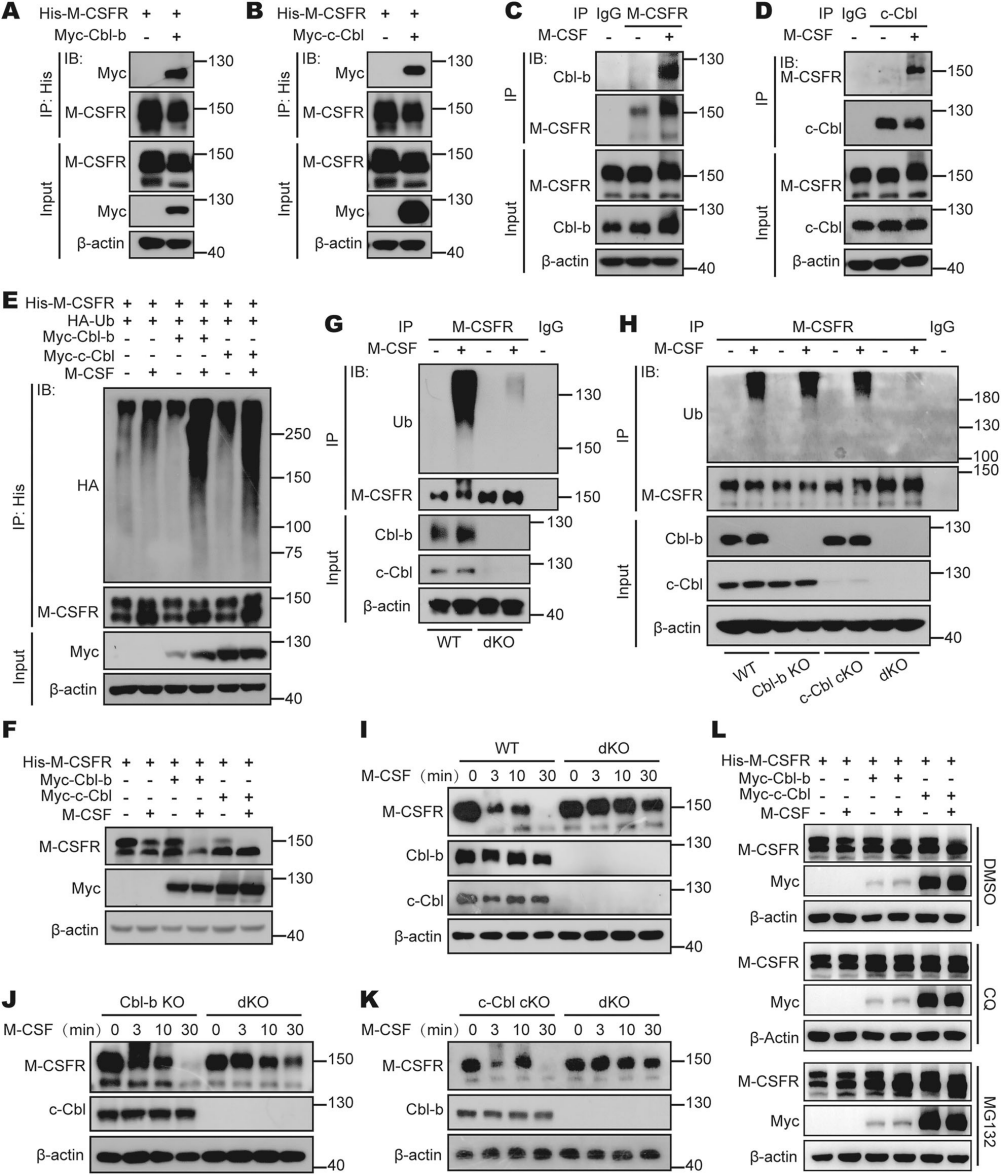
Cbls mediate the ubiquitination and subsequent degradation of M-CSFR in response to M-CSF induction. **A** Immunoprecipitation analysis of the exogenous interaction between His-M-CSFR and Myc-Cbl-b in HEK293T cells. **B** Immunoprecipitation analysis of the exogenous interaction between His-M-CSFR and Myc-c-Cbl in HEK293T cells. **C** WT BMDMs were starved of M-CSF for 24 h and restimulated with M-CSF (50 ng/mL) and harvested for immunoprecipitation analysis of the endogenous interaction between M-CSFR and Cbl-b. **D** WT BMDMs were starved of M-CSF for 24 h and restimulated with M-CSF (50 ng/mL) and harvested for immunoprecipitation analysis of the endogenous interaction between M-CSFR and c-Cbl. **E** Immunoprecipitation analysis of polyubiquitination of M-CSFR in HEK293T cells cotransfected with His-M-CSFR, HA-Ub and Myc-Cbl (Cbl-b or c-Cbl) and then treated with M-CSF (50 ng/mL) for 2 h. **F** Western blot analysis of M-CSFR in HEK293T cells cotransfected with His-M-CSFR and Myc-Cbl (Cbl-b or c-Cbl) and then treated with M-CSF (50 ng/mL) and CHX (50 µM) for 2 h. **G** WT and dKO BMDMs were starved of M-CSF for 24 h and restimulated with M-CSF (50 ng/mL) for 3 min and harvested for Co-immunoprecipitation analysis of polyubiquitination of M-CSFR. **H** WT, Cbl-b KO, c-Cbl cKO and dKO BMDMs were starved of M-CSF for 24 h and restimulated with M-CSF (50 ng/mL) for 3 min and harvested for Co-immunoprecipitation analysis of polyubiquitination of M-CSFR. **I** WT and dKO BMDMs were starved of M-CSF for 24 h and restimulated with M-CSF (50 ng/mL) for indicated times and harvested for western blot analysis of degradation of M-CSFR. **J** Cbl-b KO and dKO BMDMs were starved of M-CSF for 24 h and restimulated with M-CSF (50 ng/mL) for indicated times and harvested for western blot analysis of degradation of M-CSFR. **K** c-Cbl cKO and dKO BMDMs were starved of M-CSF for 24 h and restimulated with M-CSF (50 ng/mL) for indicated times and harvested for western blot analysis of degradation of M-CSFR. **L** Western blot analysis of M-CSFR in HEK293T cells cotransfected with His-M-CSFR and Myc-Cbl (Cbl-b or c-Cbl) and then treated with M-CSF (50 ng/mL), DMSO, CQ (20 µM) and MG132 (10 µM) for 2 h as indicated.

As members of the Cbl family are E3 ubiquitin ligases, we next investigated whether Cbls negatively regulate M-CSFR via ubiquitination and degradation. Since endogenous M-CSFR was not expressed in HEK293T cells, we constructed an overexpression plasmid of M-CSFR and confirmed that M-CSFR could be expressed on the cell membrane of HEK293T cells by Western blot and flow cytometry (Supplementary Fig. 4A, B). First, we found that exogenous M-CSFR was associated with Cbl-b or c-Cbl when they were co-expressed in HEK293T cells (Fig. 4A, B). Furthermore, endogenous M-CSFR was inducibly associated with Cbl-b or c-Cbl in MH-S cells (Supplementary Fig. 4C, D). and BMDMs (Fig. 4C, D) after stimulation with M-CSF. Additionally, we performed verification using a human cell line. THP-1 is a well-known acute monocytic leukemia cell line that exhibits similarities to primary human monocytes and differentiates into Adherent macrophages upon treatment with PMA (Supplementary Fig. 4E, F). Importantly, we observed a phenomenon identical to that seen in mouse macrophages within the PMA-stimulated THP-1 cells. Endogenous M-CSFR was found to be inducibly associated with Cbl-b or c-Cbl in PMA-stimulated THP-1 cells after stimulation with M-CSF (Supplementary Fig. 4G, H).

To investigate whether Cbls could ubiquitinate and degrade M-CSFR, we co-expressed M-CSFR with Cbl-b or c-Cbl in HEK293T cells and examined the ubiquitination and degradation of M-CSFR with or without M-CSF stimulation. Overexpression of either Cbl-b or c-Cbl significantly promoted the ubiquitination and degradation of M-CSFR after stimulation with M-CSF (Fig. 4E, F). In contrast, overexpression of either Cbl-b C373A or c-Cbl C379A, two ubiquitin E3 ligase inactive mutants of Cbl-b and c-Cbl, significantly down-regulated M-CSFR ubiquitination and prevented M-CSFR degradation (Supplementary Fig. 4I–L). To further confirm the effect of endogenous Cbl-b and c-Cbl in primary cells on the ubiquitination modification of M-CSFR, we stimulated BMDMs from WT and dKO mice with M-CSF. We found that M-CSF stimulation significantly enhanced M-CSFR ubiquitination in WT BMDMs (Fig. 4G). However, double deletion of Cbl-b and c-Cbl significantly reduced M-CSF-induced M-CSFR ubiquitination (Fig. 4G). Since the two molecules, Cbl-b and c-Cbl, co-exist in WT BMDMs, we wanted to further explore whether endogenous Cbl-b or c-Cbl can play the same role when they are present alone. Next, we stimulated BMDMs from WT, Cbl-b KO, c-Cbl KO and dKO mice with M-CSF. We found that compared with WT BMDMs, M-CSFR in Cbl-b KO and c-Cbl cKO BMDMs can still undergo significant polyubiquitination modification under M-CSF stimulation (Fig. 4H). However, M-CSFR in dKO mouse BMDMs did not undergo significant polyubiquitination modification under M-CSF stimulation (Fig. 4H). Consequently, the signal-induced degradation of M-CSFR after M-CSF stimulation was significantly blocked only in dKO BMDMs but not in Cbl-b-deficient or c-Cbl-deficient BMDMs (Fig. 4I-K). We also observed that after PMA-stimulated THP-1 cells were stimulated with M-CSF, the expression of M-CSFR decreased with the extension of stimulation time (Supplementary Fig. 4M). This indicates that the findings in mouse cells may be applicable to human cells.

To further understand the pathways by which Cbl-b and c-Cbl promote M-CSFR degradation, we treated HEK293T cells with either CQ (a lysosome inhibitor) or MG132 (a proteasome inhibitor). The results showed that lysosomal inhibition by CQ significantly blocked Cbl-b and c-Cbl-mediated M-CSFR degradation after M-CSF stimulation, whereas MG132 had no effect on proteasome inhibition (Fig. 4L). Thus, these data indicate that both Cbl-b and c-Cbl target M-CSFR for lysosomal degradation after M-CSF stimulation.

### Phosphorylation of Cbls and autophosphorylation of M-CSFR are critical for M-CSFR ubiquitination and degradation

**Fig. 5:**
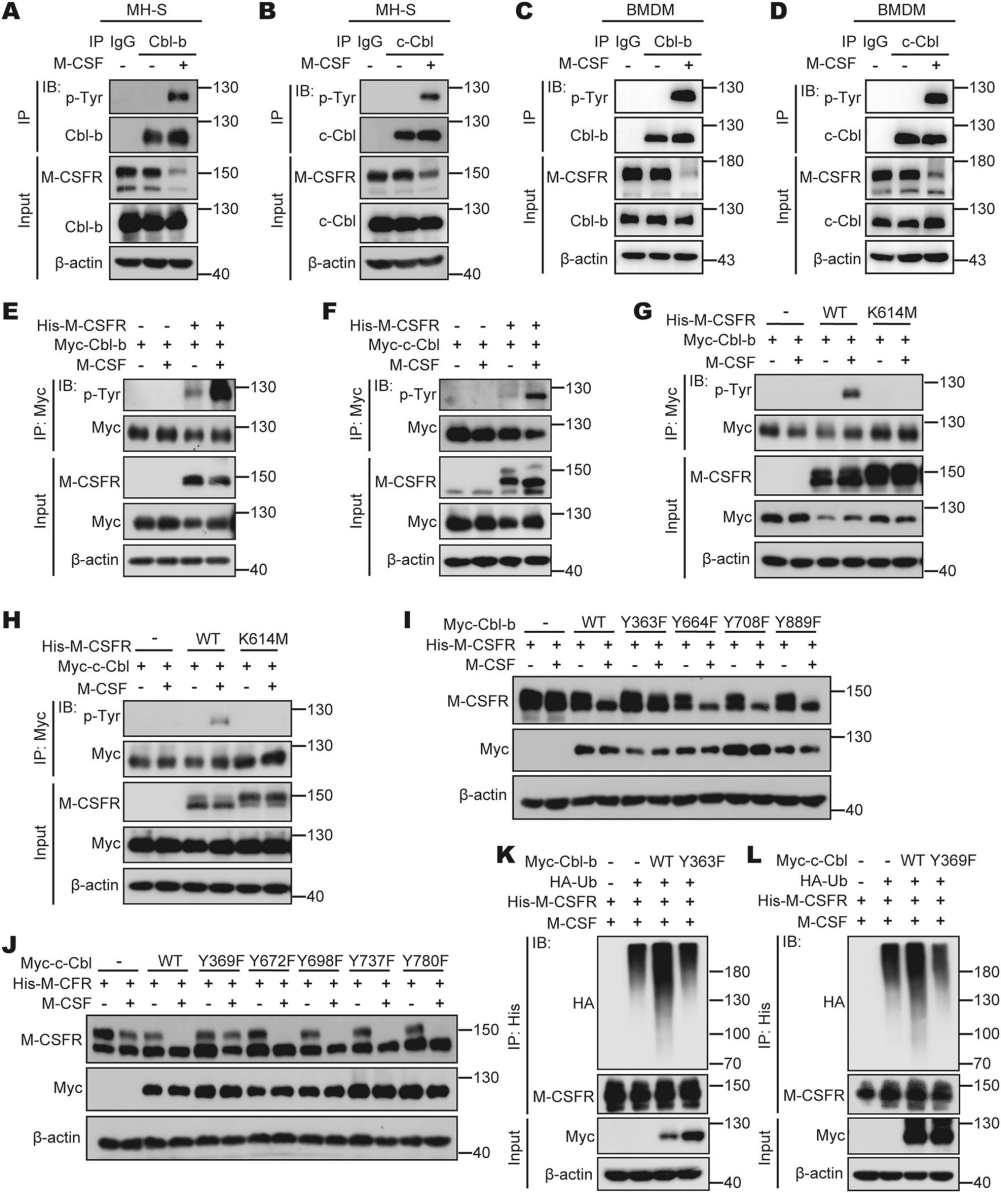
Phosphorylation of Cbls is critical for M-CSFR ubiquitination and degradation. **A** Immunoprecipitation analysis of tyrosine phosphorylation of Cbl-b in RAW 264.7 cells treated with M-CSF (50 ng/mL). **B** Immunoprecipitation analysis of tyrosine phosphorylation of c-Cbl in RAW 264.7 cells treated with M-CSF (50 ng/mL). **C** WT BMDMs were starved of M-CSF for 24 h and restimulated with M-CSF (50 ng/mL) and harvested for Immunoprecipitation analysis of tyrosine phosphorylation of Cbl-b. **D** WT BMDMs were starved of M-CSF for 24 h and restimulated with M-CSF (50 ng/mL) and harvested for Immunoprecipitation analysis of tyrosine phosphorylation of c-Cbl. **E** Immunoprecipitation analysis of tyrosine phosphorylation of Cbl-b in HEK293T cells cotransfected with Myc-Cbl-b and His-M-CSFR and then treated with M-CSF (50 ng/mL) for 3 min. **F** Immunoprecipitation analysis of tyrosine phosphorylation of c-Cbl in HEK293T cells cotransfected with Myc-c-Cbl and His-M-CSFR and then treated with M-CSF (50 ng/mL) for 3 min. **G** Immunoprecipitation analysis of tyrosine phosphorylation of Cbl-b in HEK293T cells cotransfected with Myc-Cbl-b and His-M-CSFR (WT or K614M) and then treated with M-CSF (50 ng/mL) for 3 min. **H** Immunoprecipitation analysis of tyrosine phosphorylation of c-Cbl in HEK293T cells cotransfected with Myc-c-Cbl and His-M-CSFR (WT or K614M) and then treated with M-CSF (50 ng/mL) for 3 min. **I** Western blot analysis of M-CSFR in HEK293T cells cotransfected with His-M-CSFR and Myc-Cbl-b (WT or its tyrosine mutants) and then treated with M-CSF (50 ng/mL) and CHX (50 µM) for 2 h. **J** Western blot analysis of M-CSFR in HEK293T cells cotransfected with His-M-CSFR and Myc-c-Cbl (WT or its tyrosine mutants) and then treated with M-CSF (50 ng/mL) and CHX (50 µM) for 2 h. **K** Immunoprecipitation analysis of polyubiquitination of M-CSFR in HEK293T cells cotransfected with His-M-CSFR, HA-Ub and Myc-Cbl-b (WT or Y363F) and then treated with M-CSF (50 ng/mL) for 2 h. **L** Immunoprecipitation analysis of polyubiquitination of M-CSFR in HEK293T cells cotransfected with His-M-CSFR, HA-Ub and Myc-c-Cbl (WT or Y369F) and then treated with M-CSF (50 ng/mL) for 2 h.

Studies have shown that both Cbl-b and c-Cbl contain various tyrosine residues, which can be phosphorylated under multiple stimuli, and tyrosine phosphorylation of Cbl-b and c-Cbl is critical for their biological activity [40, 41]. To further investigate whether Cbl-b and c-Cbl can undergo tyrosine phosphorylation during M-CSF stimulation, we stimulated MH-S cells or WT mice primary BMDMs with or without M-CSF. We found that M-CSF stimulation can induce prominent tyrosine phosphorylation of both Cbl-b and c-Cbl (Fig. 5A–D). Furthermore, we observed that stimulation with M-CSF induces significant tyrosine phosphorylation of both Cbl-b and c-Cbl in PMA-stimulated THP-1 cells (Supplementary Fig. 5A, B). To determine whether the tyrosine phosphorylation of Cbls is mediated by their target kinase M-CSFR, we expressed Cbls alone or with M-CSFR in HEK293T cells, and then measured the tyrosine phosphorylation levels of Cbls with or without M-CSF stimulation. The results showed that M-CSF stimulation did not induce tyrosine phosphorylation when Cbl-b or c-Cbl was expressed alone, but M-CSF stimulation significantly increased the tyrosine phosphorylation level of Cbl-b or c-Cbl when they were co-expressed with M-CSFR (Fig. 5E, F). When M-CSFR loses its kinase activity, it cannot mediate tyrosine phosphorylation of Cbl-b or c-Cbl (Fig. 5G, H). These results suggest that M-CSFR can mediate tyrosine phosphorylation of Cbl-b or c-Cbl after M-CSF stimulation, depending on the presence of M-CSFR receptor tyrosine kinase activity.

Based on previous studies of Cbls and information from the UniProt database, we noticed that Cbl-b contains four of the most significantly phosphorylated tyrosine (Tyr, F) residues (including Tyr363, 664, 708 and 889) and c-Cbl contains five of the most significantly phosphorylated tyrosine residues (Tyr369, 672, 698, 737 and 780). To further investigate whether M-CSFR-mediated tyrosine phosphorylation of Cbls during M-CSF stimulation has an effect on ubiquitination and degradation of M-CSFR, we mutated each tyrosine (Y) residue to phenylalanine (F) to simulate non-phosphorylation. Overexpression of either Cbl-b Y363F or c-Cbl Y369F in HEK293T cells did not promote the degradation of M-CSFR during M-CSF stimulation (Fig. 5I, J). Furthermore, overexpression of either Cbl-b Y363F or c-Cbl Y369F in HEK293T cells significantly down-regulated the level of M-CSFR polyubiquitination modification during M-CSF stimulation (Fig. 5K, L). These results suggest that tyrosine phosphorylation of M-CSFR at Cbl-b Tyr-363 or c-Cbl Tyr-369 mediates ubiquitination and degradation of M-CSFR by Cbl-b or c-Cbl, respectively, during M-CSF stimulation.

We also found that when the kinase activity of M-CSFR was lost, Cbls were unable to degrade the M-CSFR kinase inactivated mutant K614M during M-CSF stimulation (Supplementary Fig. 6A). Consistent with these results, overexpression of either Cbl-b or c-Cbl significantly up-regulated the polyubiquitination level of M-CSFR (WT) under M-CSF stimulation, whereas overexpression of either Cbl-b or c-Cbl did not up-regulate the polyubiquitination level of M-CSFR kinase inactivated mutant K614M (Supplementary Fig. 6B, C). These results suggest that M-CSFR kinase activity is required for M-CSFR ubiquitination and degradation through Cbls.

M-CSF binds to M-CSFR on the cell surface to dimerize M-CSFR, and dimerization activates M-CSFR tyrosine autophosphorylation and recruitment of related signaling proteins. Since M-CSFR is autophosphorylated during M-CSF stimulation, we wondered what the impact of these events is on M-CSFR stability. First, we stimulated WT BMDMs with M-CSF at different time points, and treated the cells in combination with M-CSFR inhibitor BLZ945 (BLZ945 acts as a potent and selective small molecule inhibitor of M-CSFR receptor tyrosine kinase, blocking the autophosphorylation activation of M-CSFR), and then detected the protein expression level of M-CSFR. The results showed that M-CSFR in WT BMDMs was rapidly degraded during M-CSF stimulation, and the treatment of WT BMDMs with BLZ945 effectively prevented M-CSFR degradation induced by M-CSF stimulation (Supplementary Fig. 6D, E). These results suggest that BLZ945 inhibition of autophosphorylation activation of M-CSFR can effectively prevent the degradation of M-CSFR.

Based on previous studies of M-CSFR and information from the UniProt, we noticed that M-CSFR contains eight of the most significantly phosphorylated tyrosine (Tyr, F) residues (including Tyr544, 559, 697, 706, 721, 807, 921 and 974). To examine whether autophosphorylation is required for M-CSFR ubiquitination and degradation by Cbl-b and c-Cbl, we each tyrosine (Y) residue to phenylalanine (F) to simulate non-phosphorylation and construct a series of M-CSFR phosphorylated mutant plasmids. We found that overexpression of either Cbl-b or c-Cbl effectively promoted the degradation of M-CSFR WT, Y544F, Y697F, Y706 F, Y721F, Y807F, Y921F and Y974F after stimulation with M-CSF, but not Y559F (Supplementary Fig. 6F, G). Importantly, mutation of Tyr559 abolished Cbl-b or c-Cbl-mediated ubiquitination of M-CSFR induced by M-CSF stimulation (Supplementary Fig. 6H, I). These data suggest that autophosphorylation of Tyr559 is critical for M-CSFR ubiquitination and degradation by Cbls.

### Cbls mediate the K63-linked polyubiquitination modification at Lys791 of M-CSFR

**Fig. 6:**
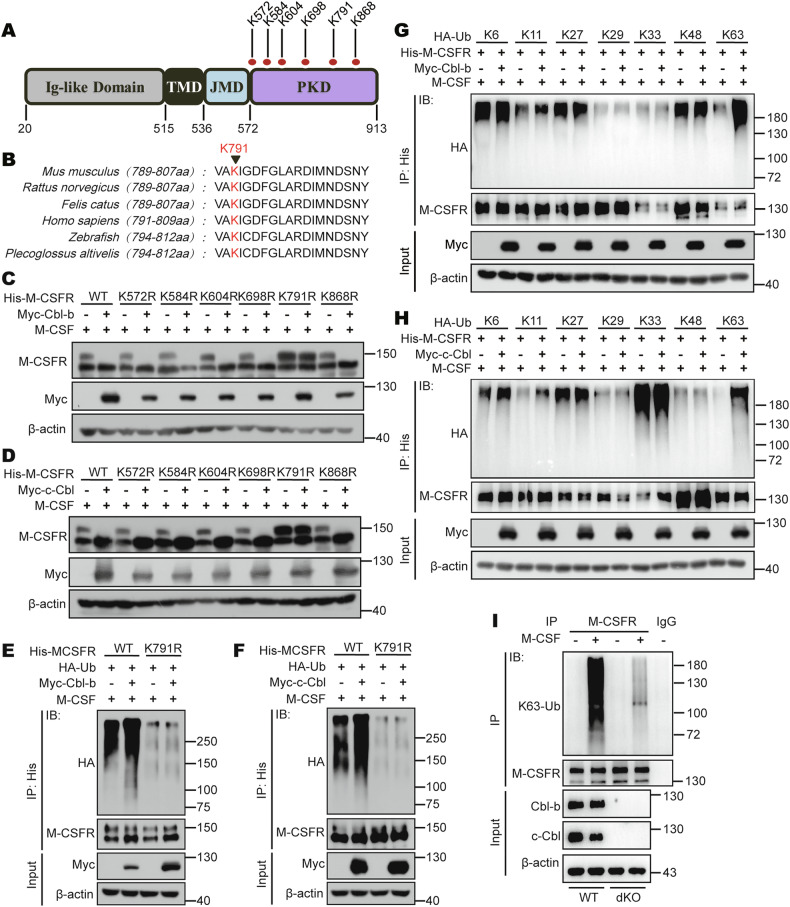
Cbls mediate the K63-linked polyubiquitination modification at Lys791 of M-CSFR. **A** Model diagram of M-CSFR structure and its potential ubiquitination site. **B** Highly conserved lysine (K) residues (K791) on M-CSFR from different species. **C** Western blot analysis of M-CSFR in HEK293T cells cotransfected with His-M-CSFR (WT or its lysine mutants) and Myc-Cbl-b and then treated with M-CSF (50 ng/mL) and CHX (50 µM) for 2 h. **D** Western blot analysis of M-CSFR in HEK293T cells cotransfected with His-M-CSFR (WT or its lysine mutants) and Myc-c-Cbl and then treated with M-CSF (50 ng/mL) and CHX (50 µM) for 2 h. **E** Immunoprecipitation analysis of polyubiquitination of M-CSFR in HEK293T cells cotransfected with His-M-CSFR (WT or K791R), HA-Ub and Myc-Cbl-b and then treated with M-CSF (50 ng/mL) for 2 h. **F** Immunoprecipitation analysis of polyubiquitination of M-CSFR in HEK293T cells cotransfected with His-M-CSFR (WT or K791R), HA-Ub and Myc-c-Cbl and then treated with M-CSF (50 ng/mL) for 2 h. **G** Immunoprecipitation analysis of polyubiquitination types of M-CSFR in HEK293T cells cotransfected with His-M-CSFR, Myc-Cbl-b and different types of HA-Ub and then treated with M-CSF (50 ng/mL) for 2 h. **H** Immunoprecipitation analysis of polyubiquitination types of M-CSFR in HEK293T cells cotransfected with His-M-CSFR, Myc-c-Cbl and different types of HA-Ub and then treated with M-CSF (50 ng/mL) for 2 h. **I** WT and dKO BMDMs were starved of M-CSF for 24 h and restimulated with M-CSF (50 ng/mL) for 3 min and harvested for Co-immunoprecipitation analysis of K63-linked polyubiquitination of M-CSFR.

We further analysed the putative lysine (Lys, K) residues of M-CSFR with ubiquitination modifications from the Protein Lysine Modifications Database (PMLD). We noticed that there are six potential ubiquitinated lysine residues on M-CSFR including Lys572, 584, 604, 698, 791, and 868 (Fig. 6A). Next, we further used the NCBI database to perform site sequence homology comparison, and the results showed that K791 was a conserved ubiquitination motif in the M-CSFR homology (Fig. 6B). This suggested that K791 might be a site of polyubiquitination modification on M-CSFR. To verify this hypothesis, we mutated each lysine (K) residue to arginine (R). We found that overexpression of either Cbl-b or c-Cbl effectively promoted the degradation of M-CSFR WT, K572R, K584R, K604R, K698R, and K868R after stimulation with M-CSF, but not K791R (Fig. 6C, D). Importantly, mutation of Lys791 abolished Cbl-b or c-Cbl-mediated ubiquitination of M-CSFR induced by M-CSF stimulation (Fig. 6E, F). These data suggest that Cbls induce polyubiquitination of M-CSFR at Lys791 during M-CSF stimulation.

To determine which type of polyubiquitin linkage to M-CSFR is catalyzed by Cbls, we transfected M-CSFR and Cbl-b or c-Cbl into HEK293T cells together with different ubiquitin mutant types of HA-Ub, including K6, K11, K27, K29, K33, K48 and K63, each of which contains only one lysine available for polyubiquitination. We found that overexpression of either Cbl-b or c-Cbl significantly promoted K63-linked polyubiquitination of M-CSFR after stimulation with M-CSF (Fig. 6G, H). Furthermore, mutation of Lys791 largely inhibited Cbl-b or c-Cbl-mediated K63-linked polyubiquitination of M-CSFR after stimulation with M-CSF (Supplementary Fig. 7A, B). At the same time, we found that M-CSF stimulation significantly enhanced M-CSFR K63-linked polyubiquitination in WT BMDMs (Fig. 6I). However, double deletion of Cbl-b and c-Cbl significantly reduced M-CSF-induced M-CSFR K63-linked polyubiquitination (Fig. 6I). Thus, we demonstrated that Cbls induce K63-linked polyubiquitination at Lys791 of M-CSFR during M-CSF stimulation, leading to M-CSFR protein endogenous degradation via the lysosomal pathway.

### The Cbls ablation alters the homeostasis of peripheral macrophages

**Fig. 7:**
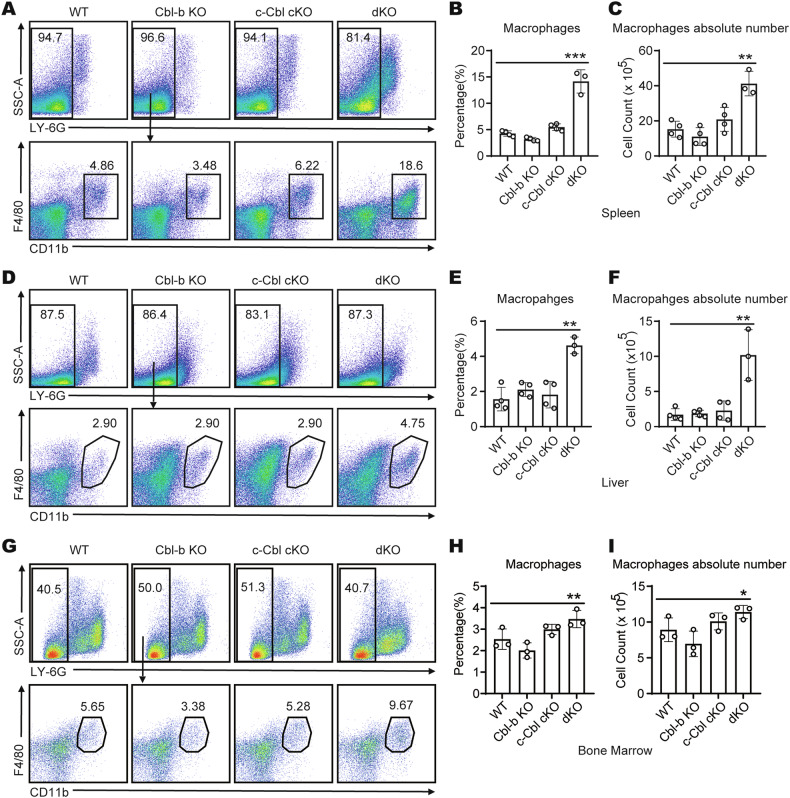
Tissue macrophages are increased in dKO mice. **A** Flow cytometry analysis of macrophages (LY-6G^-^ F4/80^+^ CD11b^+^) in spleens from four groups of mice (*n* = 3-4 per group). Statistics of percentage **B** and absolute number **C** of macrophages in spleens from four groups of mice (*n* = 3-4 per group), as shown in A. **D** Flow cytometry analysis of macrophages (LY-6G^-^ F4/80^+^ CD11b^+^) in leukocytes which were isolated from four groups of mice livers (*n* = 3-4 per group). Statistics of percentage **E** and absolute number **F** of macrophages in leukocytes which were isolated from four groups of mice livers (*n* = 3-4 per group), as shown in D. **G** Flow cytometry analysis of macrophages (LY-6G^-^ F4/80^+^ CD11b^+^) in bone marrow from four groups of mice livers (*n* = 3 per group). Statistics of percentage **H** and absolute number **I** of macrophages in bone marrow from four groups of mice livers (*n* = 3 per group), as shown in G. The “*n*” represents the number of biologically independent samples. One-Way ANOVA comparisons for **B, C, E, F, H** and **I**. *p < 0.05, **p < 0.01, ***p < 0.001. *p* < 0.05 was considered statistically significant.

**Fig. 8:**
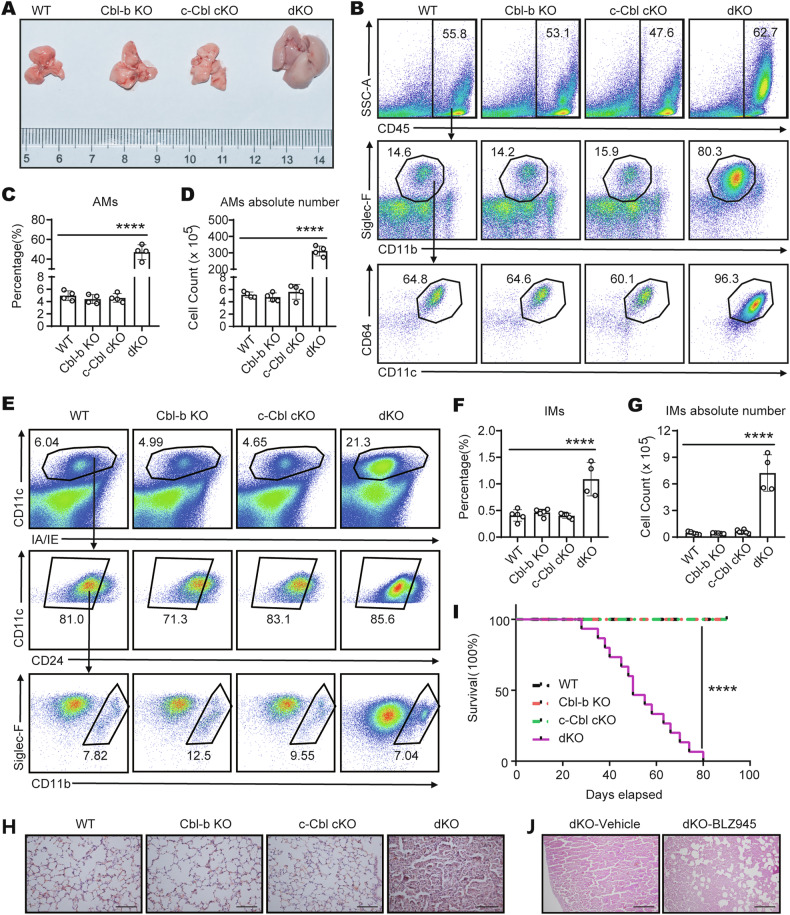
Abnormal macrophage accumulation in the lungs of dKO mice. **A** Lungs of mice of all four genotypes were dissected for morphologic examination (*n* = 3 per group). **B** Flow cytometry analysis of AMs (CD45^+^ Siglec-F^+^ CD11b^int^ CD64^+^ CD11c^+^) in lungs from four groups of mice (*n* = 4 per group). Statistics of percentage **C** and absolute number **D** of AMs in lungs from four groups of mice (*n* = 4 per group), as shown in B. **E** Flow cytometry analysis of interstitial macrophages (IMs) (CD11c^+^ IA/IE^+^ CD24^-^ Siglec-F^-^ CD11b^high^) in lungs from four groups of mice (*n* = 4 per group). Statistics of percentage **F** and absolute number **G** of IMs in lungs from four groups of mice (*n* = 4 per group), as shown in E. **H** Microscopy of hematoxylin and eosin staining of lung sections from four groups of mice (*n* = 6 per group); scale bar, 100 μm. **I** Survival curves of four groups of mice (*n* = 15 per group). **J** Microscopy of hematoxylin and eosin staining on lung sections of dKO mice (*n* = 3 per group) in the control group (Vehicle) and the administration group (BLZ945); scale bar, 200 μm. The “*n*” represents the number of biologically independent samples. One-Way ANOVA comparisons for **C, D, F** and **G**, log-rank tests for **I**. ****p < 0.0001. *p* < 0.05 was considered statistically significant.

Given the elevated proliferation and improved survival of dKO BMDMs under M-CSF stimulation in vitro, we tried to determine whether there was any notable abnormality in vivo. We compared macrophages in spleen, liver, and bone marrow of WT, Cbl-b KO, c-Cbl cKO, and dKO mice by flow staining. The results showed that the proportion and absolute number of macrophages in spleen, liver and bone marrow of dKO mice were significantly increased compared with WT, Cbl-b KO, and c-Cbl cKO mice (Fig. 7A–I). Additionally, we noted that dKO mice spontaneously developed pulmonary enlargement (Fig. 8A). Flow cytometry revealed that the proportion and absolute number of lung macrophages, including alveolar macrophages (AMs) and interstitial macrophages (IMs), were significantly increased in dKO mice compared to WT, Cbl-b KO, and c-Cbl cKO mice (Fig. 8B–G). These data indicate the role of Cbls in regulating tissue macrophage numbers in vivo.

Meanwhile, we performed H&E staining analysis on the lung tissues of the four groups of mice. The lung tissues of WT and two types of single-knockout mice exhibited normal reticular structures. In contrast, the lung tissues of dKO mice lost its normal structure, with extensive infiltration of inflammatory cells (mainly macrophages), extensive fusion thickening of alveolar walls, and the alveolar spaces almost completely obliterated (Fig. 8H). The pathological score (multi-parameter comprehensive scoring method) of H&E staining sections of lung tissues in dKO mice was significantly higher than that of the other three groups of mice (Supplementary Fig. 8A). It is well established that alveoli serve as the primary sites for gas exchange. The loss of alveolar structure results in compromised lung function in mice, which can have severe consequences for their health. Correspondingly, we observed that dKO mice exhibited symptoms such as dyspnea, lethargy, and reduced activity levels.

To further evaluate the pulmonary function of mice, we conducted a phenotypic analysis of the responses of WT and dKO mice to the stimulation of the bronchoconstrictor methacholine challenge using whole body plethysmography (WBP). Dose-response measurements were conducted using methacholine (0, 6.25, 12.5, 25 and 50 mg/ml) in the experiment. Consistent with our observation of tachypnea in dKO mice. The test results showed that the breathing frequence (f) of dKO mice was always much faster than that of WT mice (Supplementary Fig. 8B). The respiratory rate of dKO mice significantly increased, accompanied by a significant reduction in inspiratory time (Ti) (Supplementary Fig. 8C) and expiratory time (Te) (Supplementary Fig. 8D), as well as a significant decrease in tidal volume, baseline (TVb) (Supplementary Fig. 8E). Furthermore, compared with WT mice, the expiratory end pressure (EEP) of dKO mice decreased to an extremely low level (Supplementary Fig. 8F). These results undoubtedly explain the dyspnea and premature death of dKO mice (Fig. 8I).

Next, we collected bronchoalveolar lavage fluid (BALF) from mice for flow cytometry analysis. Flow cytometry revealed that, consistent with the results in lung tissue, the percentage and absolute number of AMs in dKO mice BALF significantly increased (Supplementary Fig. 9A–C). To further verify whether Cbls maintain macrophage homeostasis by regulating the M-CSF/M-CSFR pathway in vivo, we examined M-CSFR expression on the surface of AMs from WT, Cbl-b KO, c-Cbl cKO and dKO mice. Flow cytometry revealed that M-CSFR expression was significantly higher on dKO AMs compared to that on WT, Cbl-b KO and c-Cbl cKO AMs (Supplementary Fig. 9D). We also analyzed the proliferation and apoptosis of AMs. As expected, compared with WT, Cbl-b KO, and c-Cbl cKO mice, the proliferation of AMs in dKO mice significantly increased, and the apoptosis of AMs in dKO mice was greatly reduced (Supplementary Fig. 9E–G). We also treated dKO mice with the M-CSFR inhibitor BLZ945. We selected dKO mice approximately 2 weeks of age and administered either 20% Captisol® vector or 200 mg/kg BLZ945. The administration was performed via oral gavage once daily for two consecutive weeks. Following the completion of the treatment, the mice were sacrificed, and lung tissues were collected for H&E staining analysis. The result showed that administration of BLZ945, which blocks M-CSFR activation, alleviated the pulmonary pathology in dKO mice to a certain extent (Fig. 8J and Supplementary Fig. 9H). Therefore, these data indicate that the deficiency of Cbls can upregulate the expression of M-CSFR protein on the surface of macrophages, thereby promoting macrophage proliferation and inhibiting apoptosis, which contributes to the accumulation of macrophages in vivo.

## Discussion

A recent study has demonstrated that the double knockout of Cbl-b and c-Cbl enables macrophages to sustain elevated cell growth and evade senescence. This phenomenon arises from the negative regulation of M-CSFR, a critical factor in macrophage development, by Cbls. Specifically, Cbls negatively modulate M-CSFR signal transduction by facilitating the ubiquitination of M-CSFR, thereby obstructing the signals that promote macrophage proliferation. This study not only elucidates a previously underappreciated pivotal role of Cbls in macrophage biology but also provides essential foundational evidence for understanding macrophage development and homeostasis [42]. However, there remains a lack of direct evidence regarding whether Cbls sustain macrophage homeostasis (including both quantity and function) in vivo by modulating the M-CSF/M-CSFR signaling pathway. To address this research gap, we conducted relevant studies by developing a dKO mouse model. Our findings confirmed that the specific deletion of Cbls in macrophages alleviates their inhibition of the M-CSF/M-CSFR signaling pathway, resulting in sustained activation of AKT, which is the primary target of the downstream key effector molecule PI3K. This persistent activation of AKT signaling significantly enhances macrophage proliferation and inhibits apoptosis, ultimately leading to a marked increase in the number of macrophages within the spleen, liver, bone marrow, and lung tissues of dKO mice. Furthermore, dKO mice exhibit spontaneous lung enlargement characterized by macrophage proliferation, which ultimately results in respiratory difficulties and premature mortality among these animals. Treatment with BLZ945 has been shown to reduce macrophage proliferation, increase cell apoptosis, and alleviate lung pathology in dKO mice. Although BLZ945 has not yet received approval from the US FDA, its remarkable selective inhibitory capacity against M-CSFR and favorable tissue distribution potential—particularly as the only small molecule inhibitor of M-CSFR that has been clearly demonstrated to penetrate the blood-brain barrier—render it our preferred therapeutic agent [43–45]. In conclusion, our research findings have identified Cbls as key regulatory factors essential for maintaining macrophage homeostasis within tissues, and have indicated that alterations in macrophage quantity homeostasis can disrupt overall health status in the body.

Lyz2-Cre is a powerful tool for investigating the overall function of myeloid cells and is currently widely employed for the specific knockout of target genes in macrophages. However, it should be noted that it is not exclusively macrophage-specific. Lyz2 is also highly expressed in neutrophils, and the Lyz2-Cre promoter exhibits activity in monocytes and DCs [46–48]. This broader activity poses significant limitations for our research and raises concerns regarding whether the lung enlargement observed in dKO mice is primarily attributable to excessive macrophage proliferation. Our experimental data indicate that, compared to control group mice, the proportions of monocytes, CD11b^+^ conventional DCs (cDCs), and CD103^+^ cDCs were significantly reduced in in the lung tissues of dKO mice; however, their absolute numbers showed no significant changes (Supplementary Fig. 10). Our previous studies have demonstrated that the specific knockout of c-Cbl and Cbl-b in DCs using CD11c Cre does not lead to severe lung diseases in dKO mice [29]. This eliminates the substantial influence of certain CD11c+ cells, including DCs. Furthermore, both the proportions and absolute numbers of neutrophils and eosinophils in the lung tissues of dKO mice were markedly decreased (Supplementary Fig. 11). Quantitatively, the increase in macrophages within dKO lung tissue is highly pronounced. Meanwhile, the adoptive transfer experiments demonstrated that the transfer of dKO AMs or BMDMs to irradiated WT mice resulted in structural damage to the recipient lung tissue (Supplementary Fig. 12). These findings suggest that the phenotypes observed in dKO mice are significantly influenced by alterations in macrophage populations.

The absence of Cbls results in an increased number of macrophages across various tissues. We have undertaken a mechanistic exploration to investigate this phenomenon. It is worth noting that this phenomenon is particularly significant in the lung tissue of mice, accompanied by obvious morphological changes. The synergistic effects of the dual cytokines, M-CSF and GM-CSF, may offer valuable insights into this phenomenon. Studies have demonstrated that alveolar epithelial cells serve as the primary source of GM-CSF production and establish the local microenvironment essential for the survival of AMs. A deficiency in either GM-CSF or GM-CSFR signaling can result in developmental defects in AMs [49–51]. The development and homeostasis maintenance of resident macrophages in other tissues mainly depend on M-CSF, and usually not on GM-CSF. Then we assume that on the basis of the enhancement of the existing M-CSFR signaling pathway in dKO AMs, additional GM-CSF stimulation may produce a synergistic or cumulative effect, further significantly enhancing the expansion rate of these cells. Furthermore, since Lyz2-Cre is also expressed in type II alveolar epithelial cells (AT2 cells), we speculate that the absence of Cbls may also have an impact on the homeostasis of AT2 cells. In particular, we also found that the absolute number of CD45-negative cell populations in the lung tissues of dKO mice increased significantly (but to a much lower extent than that of AMs) (Supplementary Fig. 13), which might include AT2 cells. This aspect also merits further investigation in our future research endeavors.

Cbls functions as E3 ubiquitin ligase, catalyzing the ubiquitination of target proteins and thereby influencing protein stability. Typically, polyubiquitinated proteins undergo ubiquitin linkage via K48 or K63. K48-linked ubiquitination typically targets proteins for degradation by the proteasome, K63-linked ubiquitination is usually involved in protein trafficking, DNA repair, and activation of signaling pathways [52–55]. Intriguingly, our findings revealed that K63-linked ubiquitination at the K791 sites on the M-CSFR protein was critical for its degradation through the lysosomal pathway in mammalian cells. Moreover, emerging evidence also suggests that K63-linked ubiquitination is involved in lysosome-dependent degradation and plays a role in protein degradation [56–59].

Although as early as 1999, it has been reported that c-Cbl can ubiquitinate M-CSFR to promote its internalization and degradation [60]. However, the researchers found that although the M-CSF/M-CSFR complex on the surface of c-Cbl^-/-^ macrophages was internalized more slowly, it still could not avoid the complete degradation of M-CSFR, suggesting that the absence of c-Cbl alone was not sufficient to completely prevent the ubiquitination degradation of M-CSFR. This suggests that there are other factors besides c-Cbl that can regulate the degradation of M-CSFR. Huang et al. found that Cbl and Cbl-b have overlapping functions in ligand-induced M-CSFR ubiquitination, endocytosis, transport into the lumen of macropinosomes [42]. Our research results also support this finding. During the ubiquitin-mediated degradation of M-CSFR, there exists a notable redundancy between Cbl-b and c-Cbl. Furthermore, our findings indicate that the knockout of Cbl-b does not influence the expression levels of c-Cbl protein, while the knockout of c-Cbl similarly does not affect the expression levels of Cbl-b protein (Supplementary Fig. 1B). This eliminates the possibility of a compensatory role for the Cbl-b and c-Cbl. Although these studies reported M-CSFR ubiquitination of Cbl-b or c-Cbl, they did not distinguish the specific Ub linkage types and the ubiquitination sites present on activated M-CSFR. Our results expand on these studies by demonstrating that Cbl E3 ligases modify activated M-CSFR predominantly with K63 Ub linkages at Lys791 that target the kinase for degradation.

In summary, our findings uncover Cbls as important negative regulators of macrophage proliferation and positive regulators of macrophage apoptosis, and delineate a molecular framework for Cbls function in macrophages (**Graphical Abstract**). And the present study provides the first in vivo evidence to indicate important physiological roles of Cbls in regulating macrophage homeostasis. Numerous small molecule natural products have been developed as agonist analogues to enhance the expression of E3 ubiquitin ligase, making important contributions to the development of novel drugs for various diseases [61–63]. This approach is expected to provide innovative perspectives for the development of drugs to treat diseases caused by abnormal accumulation of macrophages.

## Materials and methods

### Ethics statement

All of the animal experiments were conducted in compliance with the Institutional Animal Care and Use Committee guidelines and approved by the Ethics Committee of Soochow University (Suzhou, China) (SUDA20250523A01).

### Mice

Mice were C57BL/6 background. Cbl-b^−/−^ and c-Cbl^flox/flox^ mice were kindly gifted by Dr. Hua Gu (Montreal Clinic Research Institute, Montreal, Quebec, Canada) [23]. Lyzs-Cre mice were gifted by *Prof*. Yulong He (Soochow University, China). Lyzs-Cre mice were crossed with c-Cbl^flox/flox^ to generate c-Cbl^flox/flox^ Lyzs-Cre^+^ mice. Subsequently, the progeny was crossed with Cbl-b^−/−^ mice to generate Cbl-b^−/−^ c-Cbl^flox/flox^ Lyzs-Cre^+^ (dKO) mice. All mice were housed under specific pathogen-free conditions at the Soochow University animal facility. 6–8 weeks old mice were used unless indicated with no sex-selective preference. Our study examined male and female animals, and similar findings are reported for both sexes.

### Cell culture and transfection

Human embryonic kidney (HEK) 293 T, MH-S (Mouse alveolar macrophage cell line) and THP-1 (Human acute monocytic leukemia cell line) cells were purchased from the American Type Culture Collection (ATCC). HEK293T cells cultured in DMEM medium (HyClone, Logan, Utah) containing 10% fetal bovine serum (Gibco, Hong Kong), 100 U/mL penicillin and 100 µg/mL streptomycin (Gibco, Hong Kong). MH-S and THP-1 cells cultured in RPMI 1640 medium (HyClone, Logan, Utah) containing 10% (vol/vol) fetal bovine serum (Gibco, Hong Kong), 100 U/mL penicillin and 100 µg/mL streptomycin (Gibco, Hong Kong). All cells were cultured at 37 °C under 5% CO_2_. All transient transfections were carried out using D-porzai (Merit, Tianjin, China) or PEI MAX40K (2765, Kyfora bio by Polysciences, USA) according to manufacturer’s instruction.

### Cell staining and flow cytometry

Cells were stained with fluorescence-coupled anti-mouse antibodies listed below diluted in 1 × PBS for 30 min at 4 °C and subsequently washed with 1 × PBS. Analysis was carried out on a BD FACSCanto^TM^ Ⅱ (BD Pharmingen™, USA) while cell sorting was conducted on a BD FACSAria^TM^ Ⅲ (BD Pharmingen™, USA). APC/Cy7-anti-CD11c (N418) (#117324), APC-anti-CD11c (N418) (#117310), PE/Cy7-anti-CD11c (N418) (#117318), PE-anti-CD11b (M1/70) (#101208), FITC-anti-CD11b (M1/70) (#101206), APC-anti-CD45 (30-F11) (#103112), PE-anti-CD64 (X54-5/7.1) (#139304), PerCP/Cy5.5-anti-Siglec-F (S17007L) (#155526), APC-anti-CD24 (clone M1/69) (#101814), FITC-anti-IA/IE (M5/114.15.2) (#107606), PE/Cy7-anti-IA/IE (M5/114.15.2) (#107630), PE-anti-F4/80 (BM8) (#123110), PE/Cy7-anti-Ly-6G (1A8) (#127618), PerCP/Cy5.5-anti-Ly-6G (1A8) (#127616), PE-anti-CD115 (AFS98) (#135506) were purchased from BioLegend (San Diego, USA). PE/Cy7-anti-CD11b (M1/70) (#552850), APC-anti-F4/80 (BM8) (#123116) and PE/Cy7-anti-CD3e (145-2C11) (#100220) were purchased from BD Pharmingen™ (USA).

### Cell proliferation assay

For BrdU incorporation analysis in vivo, mice aged 6-8 weeks were labeled by two intraperitoneal injections of BrdU. According to the standard of 200 mg/kg, mice were intraperitoneally injected with 200 μL of 10 mg/mL BrdU solution (Sigma-Aldrich, Germany) at 12 h and 4 h before sacrifice. AMs were isolated by BAL as“Isolation of alveolar macrophages”described. The AMs were seeded at a density of 1 × 10^4^ cells/well in a 96-well plate (NEST, Wuxi, China) and incubated for 2 h at 37 °C. The BrdU Cell Proliferation Assay Kit (CST, Danvers, MA, USA) used an anti-BrdU antibody to detect BrdU incorporated into cellular DNA during cell proliferation and the assay was performed according to the manufacturer’s instructions. The OD values at 450 nm were detected using Synergy™ H4 (BIO-TEK, USA).

For BrdU incorporation analysis in vitro, M-CSF-stimulated cells were incubated in RPMI-1640 medium containing 10 µM BrdU (Sigma-Aldrich, Germany) for 4-6 h. The Cells were stained with corresponding antibodies against surface markers and subsequently washed with 1 × PBS. Next, the cells were treated with Cytofix/Cytoperm Fixation and Permeabilization Solution (BD Pharmingen™, USA) overnight at 4 °C, digested by DNase I (Solarbio, Beijing, China) for 1 h, intracellular stained with FITC-anti-BRDU (#51-33284X) (BD Pharmingen™, USA) or APC-anti-BrdU (Bu20a) (#339808) (BioLegend, USA) for 30 min at 4 °C. After washing with 1 × BD Perm/Wash Buffer (BD Pharmingen™, USA), the cells were run on a BD FACSCanto^TM^ Ⅱ (BD Pharmingen™, USA) and data were analyzed with FlowJo software (Treestar, USA).

### Cell apoptosis assay

For the apoptosis analysis, Alveolar macrophages and M-CSF-stimulated cells were first stained with corresponding antibodies against surface markers and subsequently washed with 1 × Annexin V binding buffer (BD Pharmingen™, USA). Next, single cell suspensions were stained with APC-anti-Annexin V (#640941) (Biolegend, USA) or FITC-anti-Annexin V (#51-65874X) (BD Pharmingen™, USA) and PerCP/Cy5.5-anti-7-AAD (#51-68981E) (BD Pharmingen™, USA) in a 1 × Annexin V binding buffer according to the manufacturer’s instruction. Cells were analyzed using a BD FACSCantoTM Ⅱ (BD Pharmingen™, USA). FACS data was quantified using the FlowJo software (Tree Star, USA).

### Preparation of BMDMs and cell culture

Femur and tibia were harvested from mice, washed with 1 × PBS and bone marrow cells were flushed out with 1 × PBS. Erythrocytes were removed using ACK lysis buffer (150 mM NH4Cl, 10 mM KHCO3, 0.1 mM Na2EDTA, pH 7.2-7.4). The cell suspension was filtered through 70 μm cell strainer to remove any cell clumps and impurities. The single cell suspension was then cultured at a density of 2 × 10^6^ cells/mL in RPMI-1640 medium (HyClone, Logan, Utah) containing 10% (vol/vol) fetal bovine serum (Gibco, Hong Kong), 100 U/mL penicillin, 100 µg/mL streptomycin (Gibco, Hong Kong) and 20 ng/mL recombinant murine M-CSF (Peprotech, USA). Fresh medium containing M-CSF was added on day 3, half of the medium containing M-CSF was exchanged on day 5, and the fully differentiated BMDMs were harvested on day 7 for function assay. For M-CSFR inhibitor treatment, different concentrations of the M-CSFR inhibitor BLZ945 (Sotuletinib, MedChemExpress, USA) were added to the culture system for 7 d. The inhibitor was replenished with media exchanges.

### Western blotting

Cell lysates were obtained from cultured cells using NP-40 lysis buffer (Beyotime, Shanghai, China) containing 1 mM protease inhibitors PMSF (Beyotime, Shanghai, China) for 20-30 min on ice. The BCA Protein Assay Kit (Thermo Fisher Scientific, USA) was used to quantify protein concentration in the samples. Prior to loading, 5 × SDS loading buffer (NCM Biotech, Suzhou, China) was added to protein lysates and the samples were boiled for 10 min. Equivalent amount of proteins were subjected to SDS-PAGE gel and then were transferred to PVDF membranes (Millipore, Germany). Membranes were blocked with 5% nonfat milk (Bio Basic, Shanghai, China) or 2% BSA for 1 h at room temperature, and then incubated with the primary antibodies overnight at 4 °C. After washing three times with 0.1% PBST (1 × PBS and 0.1% Tween 20), the membranes were subjected to secondary antibodies (HRP-conjugated Affinipure Goat Anti-Mouse IgG [Proteintech, USA] or Goat Anti-Rabbit IgG-HRP [Sourthern Biotech, USA]) in 5% nonfat milk (Bio Basic, Shanghai, China) for 1 h at room temperature. After washing three times with 0.1% PBST, the membranes were treated with enhanced chemiluminescence (ECL) reagents (Bio-Rad, USA), and protein bands were observed by exposure to X-ray films (FUJIFILM, Japan). Alternatively, the immunoreactive bands were visualized under Tanon 5200 Chemiluminescence Image Analysis System (Tanon Science &Technology, Shanghai, China) with Western ECL substrate (FDbio science, Hangzhou, China). The following antibodies (Clone) with dilutions were used in our study: anti-Cbl-b (D3C12) (#9498, 1:1000), anti-c-Cbl (D4E10) (#8447, 1:1000), anti-M-CSFR (E6W9F) (#43390, 1:1000), anti-Myc (9B11) (#2276, 1:1000), anti-His (D3I1O) (#12698, 1;1000), anti-AKT (C67E7) (#4691, 1:1000), anti-p-AKT (D7F10) (#9018, 1:1000), anti-Erk1/2 (137F5) (#4695, 1:1000), anti-p-Erk (D13.14.4E) (#4370, 1:1000), anti-Phospho-Tyrosine (4G10) (#96215, 1:1000), anti-Caspase-9 (#9504, 1:1000), anti-Caspase-8 (#4927, 1:1000), anti-Caspase-7 (#9492, 1:1000), anti-Cleaved Caspase-7 (Asp198) (#9491, 1:1000), anti-Caspase-3 (D3R6Y) (#14220, 1:1000), anti-Cleaved Caspase-3 (5A1E) (#9664, 1:1000), anti-Bim (C34C5) (#2933, 1:1000), anti-Bcl-xL (54H6) (#2764, 1:1000), anti-K63-linkage Specific Polyubiquitin (D7A11) (#5261, 1:1000) and anti-Puma (E2P7G) (#98672, 1:1000), all antibodies purchased from Cell Signaling Technology (Danvers, MA, USA). Anti-Ubiquitin (P4D1) (#G2618, 1:1000) antibody was purchased from Santa Cruz (USA). Anti-HA (#T0050, 1:1000) and anti-β-Actin (#T0022, 1:5000) antibodies were purchased from Affinity Biosciences (USA).

### Immunoprecipitation

Cells were harvested in NP-40 lysis buffer (Beyotime, Shanghai, China) containing 1 mM protease inhibitors PMSF (Beyotime, Shanghai, China). When protein ubiquitination was examined, 10 mM N-ethylmaleimide (MedChemExpress, USA) was added into the NP-40 lysis buffer. When protein phosphorylation was examined, 1 × phosphatase inhibitor (Beyotime, Shanghai, China) was added into the NP-40 lysis buffer. The cell lysates were incubated with corresponding specific antibodies overnight on a rotor at 4 °C. Protein G-Agarose beads (Roche Diagnostics GmbH, Mannheim, Germany) were washed thrice with NP-40 wash buffer (5 M NaCl, 1 M Tris-HCl, pH 7.4, 1% Noidet P-40) and then were added into the supernatant. The mixture was incubated for 4-6 h on a rotor at 4 °C. Immunoprecipitates were washed once with NP-40 wash buffer, once with High Salt buffer (5 M NaCl, 0.05% Triton X-100, 1 × PBS), and finally once with NP-40 washing buffer. After boiling with 60 µL loading buffer (NP-40 wash buffer, 5× SDS loading buffe) for 10 min, the immunoprecipitates were analyzed by western blot.

### M-CSFR ubiquitination and degradation assays

For in vitro M-CSFR ubiquitination and degradation assays, HEK293T cells transfected with indicated overexpressed plasmids. 24 h later, transfected cells were treated with or without 50 ng/ml M-CSF at 37 °C for 2 h. For protein degradation pathway assay, 10 µM MG132 (Sigma-Aldrich, USA) and 20 µM CQ (MedChemExpress, USA) were used to block protein degradation, respectively. For protein ubiquitination assay, protein degradation was blocked with 20 µM CQ (MedChemExpress, USA) for 15 min before stimulation with M-CSF. Exogenous M-CSFR ubiquitination and expression were then analyzed by Imunoprecipitation and Western blot analysis using indicated antibodies.

For in vivo M-CSFR degradation assays, the fully differentiated BMDMs were incubated at 37 °C without M-CSF for 16-24 h. Cells were then stimulated with or without 50 ng/ml M-CSF at 37 °C for indicated time. For in vivo M-CSFR ubiquitination assays, the fully differentiated BMDMs were incubated at 37 °C without M-CSF for 16-24 h. Next, BMDMs stimulated with or without 50 ng/ml M-CSF at 37 °C for 1-3 min and M-CSFR degradation was blocked with 20 µM CQ for 15 min before stimulation with M-CSF. Endogenous M-CSFR ubiquitination and expression were then analyzed by Imunoprecipitation and Western blot analysis using indicated antibodies.

### Cbls phosphorylation assays

For in vivo Cbls phosphorylation assays, the fully differentiated BMDMs were incubated at 37 °C without M-CSF for 16-24 h. Next, BMDMs stimulated with or without 50 ng/ml M-CSF at 4 °C for 30 min. Endogenous Cbls phosphorylation were then analyzed by Imunoprecipitation and Western blot analysis using indicated antibodies. MH-S and THP-1 cells do not need to undergo M-CSF starvation treatment; all other treatment methods are consistent with those employed for BMDM.

For in vitro Cbls phosphorylation assays, HEK293T cells transfected with indicated overexpressed plasmids. 24 h later, transfected cells were treated with or without 50 ng/ml M-CSF at 4 °C for 30 min. Exogenous Cbls phosphorylation were then analyzed by Imunoprecipitation and Western blot analysis using indicated antibodies.

### Real-time PCR

Total RNA was extracted from BMDMs using the RNA isolater Total RNA Extraction Reagent (Vazyme, Nanjing, China). First-strand complementary DNAs (cDNAs) were synthesized using the HiScript III RT SuperMix for qPCR ( + gDNA wiper) (YEASEN, Shanghai, China). Quantitative real-time PCR was run on a QuantStudio 1 real-time fluorescence quantitative PCR system (Thermo Fisher Scientific, USA) using Hieff® qPCR SYBR® Green Master Mix (YEASEN, Shanghai, China). Quantification of all target genes was carried out by normalizing to the control gene 18S. The sequences of primers used in Real-time PCR are listed in Table S1. All primers were synthesized by Azenta (GENEWIZ, Suzhou, China).

### Preparation of mouse lung tissue single-cell suspension

Mouse lungs were cut into fine pieces and digested with 0.5 mg/mL collagenase IV (Sigma-Aldrich, USA) and 20 ng/mL DNase I (Solarbio, Beijing, China) in RPMI-1640 medium (HyClone, Logan, Utah) for 1 h at 37 °C. The cells were then washed twice with ice-cold 1 × PBS (PROLEADER, Shanghai, China) and erythrocytes were removed using ACK lysis buffer (150 mM NH4Cl, 10 mM KHCO3, 0.1 mM Na2EDTA, pH 7.2-7.4).

### Isolation of mouse liver lymphocytes

Mouse livers were cut into fine pieces and digested with 0.5 mg/mL collagenase IV (Sigma-Aldrich, USA) and 20 ng/mL DNase I (Solarbio, Beijing, China) in RPMI-1640 medium (HyClone, Logan, Utah) for 30 min at 37 °C. The cells were then washed twice with ice-cold 1 × PBS and resuspended in 10 mL Mouse 1 × Lymphocyte Separation Medium (DAKEWE, Shenzhen, China). Lymphocytes were separated according to the manufacturer’s protocol.

### Histopathology

For histopathology analysis, Lung tissues were fixed more than 24 h in a 4% paraformaldehyde solution (Solarbio, Beijing, China), processed, and then embedded in paraffin (Leica, Germany) according to standard procedures. Next, 5 μm sections were stained with hematoxylin solution (Sigma-Aldrich, USA) for 5-10 min and 0.5% eosin solution (Solarbio, Beijing, China) for 30-90 sec. The Stained sections were scanned using a microscope (ECLIPSE 90i, Nikon, Japan).

### Bronchoalveolar lavage of mice

After euthanized, the bronchoalveolar lavage (BAL) was performed on the whole lungs of mice by 2 ml 1 × PBS (PROLEADER, Shanghai, China) for 6 times with gentle massage. Almost 90% of fluid were recovered. The bronchoalveolar lavage fluid (BALF) was centrifuged at 1000 g for 5 min at 4 °C. The sediments were collected for cell counts and flow cytometry assay.

### Plasmids and reagents

HA ubiquitin (HA-Ub), HA-K6 (all lysines on the ubiquitin gene are mutated to arginines except 6), HA-K11 (all lysines on the ubiquitin gene are mutated to arginines except 11), HA-K27 (all lysines on the ubiquitin gene are mutated to arginines except 27), HA-K29 (all lysines on the ubiquitin gene are mutated to arginines except 29), HA-K33 (all lysines on the ubiquitin gene are mutated to arginines except 33), HA-K48 (all lysines on the ubiquitin gene are mutated to arginines except 48) and HA-K63(all lysines on the ubiquitin gene are mutated to arginines except 63) plasmids were gifts from Dr. Hui Zheng of Institutes of Biology and Medical Sciences (Soochow University, Suzhou, China).

The Cbl-b and c-Cbl coding sequences were amplified from the thymus cDNA of C57BL/6 J mice using 2 × Phanta Max Master Mix (Vazyme, Nanjing, China) and cloned between the KpnI and XhoI sites of the pcDNA3.1+ basic vector. The M-CSFR coding sequence was amplified from the spleen cDNA of C57BL/6 J mice using 2 × Phanta Max Master Mix and cloned into the pcDNA3.1- basic vector between the EcoRI and XhoI sites. The primer sequences used for cloning are shown in Table S2.

Five Cbl-b (C373A, Y363F, Y664F, Y708F and Y889F), six c-Cbl (C379A, Y369F, Y672F, Y698F, Y737F and Y780F) and fifteen M-CSFR (K614M, Y544F, Y559F, Y697F, Y706F, Y721F, Y807F, Y921F, Y974F, K572R, K584R, K604R, K698R, K791R and K868R) mutations were generated using the 2 × Phanta Flash Master Mix (Vazyme, Nanjing, China) and FastDigest DpnI (Thermo Fisher Scientific, Lithuania) according to the manufacturer’s instructions. Primers used for Cbl-b and c-Cbl mutations are shown in Table S3 and primers used for M-CSFR mutations are shown in Table S4.

### Retroviral packaging and infection

First of all, the Cbl-b and c-Cbl coding sequences were amplified from the thymus cDNA of C57BL/6 J mice using 2 × Phanta Max Master Mix (Vazyme, Nanjing, China) and cloned between the Xho I and HindIII sites of the pMSGV-Thy1.1 vector. Primer sequences are listed in Table S2.

Retrovirus was produced by transfecting 4 μg of each pMSGV retroviral plasmids in combination with 4 μg of helper plasmid pCL-Eco retrovirus packaging vector into 10 cm dishes with cultured HEK293T cells using PEI MAX40K (2765, Kyfora bio by Polysciences, USA). Following 48 and 72 h, the culture supernatant containing retrovirus was collected through 0.45-μm filters. Spin infection of bone marrow cells using retrovirus supernatant was performed at 2500 rpm for 90 min at 37 °C. Add 15% fetal bovine serum (Gibco, Hong Kong), 20 ng/mL recombinant murine M-CSF (Peprotech, USA) and 10 μg/mL polybrene (Sigma-Aldrich, Germany) to the retrovirus supernatant used for infection. After allowing the cells to be infected for a duration of 16 to 18 h, discard the retroviral supernatant and add fresh complete RPMI-1640 medium (containing 20ng/mL M-CSF) to continue cell culture.

### PMA cell induction assay

Firstly, the cell seeding density was fixed at 1×10^5^ cells/ml, and the cells were seeded in 12-well plates. After 24 h, THP-1 cells were stimulated with 100 ng/mL PMA. Cells were harvested at 24 h after stimulation. The cell differentiation state was observed under light microscope. And then flow cytometry was used to detect cells.

### Whole body plethysmography, WBP

6-8-week-old WT and dKO mice were selected for the experiment. All mice in the Buxco^®^ FinePointe^TM^ (DSI, USA) instrument chamber were exposed to 0, 6.25, 12.5, 25, and 50 mg/ml (i.n.) Methacholine chloride (62-51-1, MedChemExpress, USA) for 5 min. And record the pulmonary function for 5 min. Some parameters in pulmonary function tests were evaluated using instrument software.

### M-CSFR-signaling antagonist pharmacological study in dKO mice model

Select dKO mice approximately 2 weeks of age and administer either 20% Captisol® vector or 200 mg/kg BLZ945 (Sotuletinib) (953769-46-5, MedChemExpress, USA). The administration is performed via oral gavage once daily for two consecutive weeks. Following the completion of the treatment, the mice were sacrificed, and lung tissues were collected for H&E staining analysis.

### Isolation of alveolar macrophages (AMs)

AMs were isolated by BALF. BALF was collected and then centrifuged at 1000 g for 5 min at 4 °C. The cells were re-suspended with DMEM medium (HyClone, Logan, Utah) containing 10% (vol/vol) fetal bovine serum (Gibco, Hong Kong), 100 U/mL penicillin and 100 µg/mL streptomycin (Gibco, Hong Kong) and inocubated in 10 cm cell culture dishes (NEST, Wuxi, China) for 2–4 h at 37 °C. The cells adhering to the bottom of dish were collected for further experimental use.

### Adoptive transfer mouse model

In the AMs adoptive transfer experiment, 6–8 weeks old WT mice in a C57BL/6 background were irradiated and injected intravenously with BALF AMs purified from WT or dKO mice (2 × 10^6^ cells). Injections were carried out 3 times, once every 5 days. After two weeks, mice were sacrificed to assess pathologic lung changes via Hematoxylin and eosin (H&E) staining.

In the BMDMs adoptive transfer experiment, 6-8 weeks old WT mice in a C57BL/6 background were irradiated and injected intravenously with BMDMs induced from the bone marrow of WT or dKO mice (2 × 10^6^ cells). Injections were carried out 4 times, once every 5 days. After three weeks, mice were sacrificed to assess pathologic lung changes via Hematoxylin and eosin (H&E) staining.

### Statistical analysis

Statistical analyses were applied to biologically independent mice or technical replicates for each experiment. Biological replicates were performed for all experiments as indicated. Prism 8 (GraphPad) was used for statistical analysis. To assess the statistical significance of different treatment groups, we used One-Way ANOVA comparisons or unpaired two-tailed Student’s *t* tests in two different treatments. For survival curve analysis, log-rank tests were performed. *indicated *p* < 0.05; **p < 0.01; ***p < 0.001, ****p < 0.0001, ns indicates not significant. *p* values less than 0.05 were considered statistically significant. Data represent three independent experiments unless indicated.

## Supplementary information

Supplemental Material

## Data Availability

All data generated or analyzed during this study are included in this published article and its supplementary information files.

## References

[1] Martinez FO, Helming L, Gordon S. Alternative activation of macrophages: an immunologic functional perspective. Annu Rev Immunol. 2009;27:451–83. 19105661 10.1146/annurev.immunol.021908.132532

[2] Epelman S, Lavine KJ, Randolph GJ. Origin and functions of tissue macrophages. Immunity. 2014;41:21–35. 25035951 10.1016/j.immuni.2014.06.013 PMC4470379

[3] Peiró T, Patel DF, Akthar S, Gregory LG, Pyle CJ, Harker JA, et al. Neutrophils drive alveolar macrophage IL-1β release during respiratory viral infection. Thorax. 2018;73:546–56. 29079611 10.1136/thoraxjnl-2017-210010 PMC5969338

[4] Li F, Okreglicka KM, Piattini F, Pohlmeier LM, Schneider C, Kopf M. Gene therapy of Csf2ra deficiency in mouse fetal monocyte precursors restores alveolar macrophage development and function. JCI insight. 2022;7:e152271. 10.1172/jci.insight.152271 PMC9057586 35393945

[5] Kopf M, Schneider C, Nobs SP. The development and function of lung-resident macrophages and dendritic cells. Nat Immunol. 2015;16:36–44. 25521683 10.1038/ni.3052

[6] Bhargava A, Vagela M, Lennox CM. Challenges in the management of fractures in osteopetrosis”! Review of literature and technical tips learned from long-term management of seven patients. Injury. 2009;40:1167–71. 19576583 10.1016/j.injury.2009.02.009

[7] Strickland JP, Berry DJ. Total joint arthroplasty in patients with osteopetrosis: a report of 5 cases and review of the literature. J Arthroplast. 2005;20:815–20. 10.1016/j.arth.2004.11.015 16139724

[8] Hamilton JA. Colony-stimulating factors in inflammation and autoimmunity. Nat Rev Immunol. 2008;8:533–44. 18551128 10.1038/nri2356

[9] Chitu V, Stanley ER. Colony-stimulating factor-1 in immunity and inflammation. Curr Opin Immunol. 2006;18:39–48. 16337366 10.1016/j.coi.2005.11.006

[10] Stanley ER, Berg KL, Einstein DB, Lee PS, Pixley FJ, Wang Y, et al. Biology and action of colony-stimulating factor-1. Mol Reprod Dev. 1997;46:4–10. 8981357 10.1002/(SICI)1098-2795(199701)46:1<4::AID-MRD2>3.0.CO;2-V

[11] Wiktor-Jedrzejczak W, Ratajczak MZ, Ptasznik A, Sell KW, Ahmed-Ansari A, Ostertag W. CSF-1 deficiency in the op/op mouse has differential effects on macrophage populations and differentiation stages. Exp Hematol. 1992;20:1004–10. 1505635

[12] Hamilton JA. CSF-1 signal transduction. J Leukoc Biol. 1997;62:145–55. 9261328 10.1002/jlb.62.2.145

[13] Pixley FJ, Stanley ER. CSF-1 regulation of the wandering macrophage: complexity in action. Trends Cell Biol. 2004;14:628–38. 15519852 10.1016/j.tcb.2004.09.016

[14] Lee AW, States DJ. Colony-stimulating factor-1 requires PI3-kinase-mediated metabolism for proliferation and survival in myeloid cells. Cell Death Differ. 2006;13:1900–14. 16514418 10.1038/sj.cdd.4401884

[15] Smith JL, Schaffner AE, Hofmeister JK, Hartman M, Wei G, Forsthoefel D, et al. ets-2 is a target for an akt (Protein kinase B)/jun N-terminal kinase signaling pathway in macrophages of motheaten-viable mutant mice. Mol Cell Biol. 2000;20:8026–34. 11027273 10.1128/mcb.20.21.8026-8034.2000 PMC86413

[16] Lee AW. Synergistic activation of mitogen-activated protein kinase by cyclic AMP and myeloid growth factors opposes cyclic AMP’s growth-inhibitory effects. Blood. 1999;93:537–53. 9885215

[17] Rao N, Dodge I, Band H. The Cbl family of ubiquitin ligases: critical negative regulators of tyrosine kinase signaling in the immune system. J Leukoc Biol. 2002;71:753–63. 11994499

[18] Thien CB, Langdon WY. Cbl: many adaptations to regulate protein tyrosine kinases. Nat Rev Mol Cell Biol. 2001;2:294–307. 11283727 10.1038/35067100

[19] Keane MM, Rivero-Lezcano OM, Mitchell JA, Robbins KC, Lipkowitz S. Cloning and characterization of cbl-b: a SH3 binding protein with homology to the c-cbl proto-oncogene. Oncogene. 1995;10:2367–77. 7784085

[20] Keane MM, Ettenberg SA, Nau MM, Banerjee P, Cuello M, Penninger J, et al. cbl-3: a new mammalian cbl family protein. Oncogene. 1999;18:3365–75. 10362357 10.1038/sj.onc.1202753

[21] Jeon MS, Atfield A, Venuprasad K, Krawczyk C, Sarao R, Elly C, et al. Essential role of the E3 ubiquitin ligase Cbl-b in T cell anergy induction. Immunity. 2004;21:167–77. 15308098 10.1016/j.immuni.2004.07.013

[22] Naramura M, Jang IK, Kole H, Huang F, Haines D, Gu H. c-Cbl and Cbl-b regulate T cell responsiveness by promoting ligand-induced TCR down-modulation. Nat Immunol. 2002;3:1192–9. 12415267 10.1038/ni855

[23] Li X, Gadzinsky A, Gong L, Tong H, Calderon V, Li Y, et al. Cbl Ubiquitin Ligases Control B Cell Exit from the Germinal-Center Reaction. Immunity. 2018;48:530–41.e6. 29562201 10.1016/j.immuni.2018.03.006

[24] Kitaura Y, Jang IK, Wang Y, Han YC, Inazu T, Cadera EJ, et al. Control of the B cell-intrinsic tolerance programs by ubiquitin ligases Cbl and Cbl-b. Immunity. 2007;26:567–78. 17493844 10.1016/j.immuni.2007.03.015 PMC1948079

[25] Chiou SH, Shahi P, Wagner RT, Hu H, Lapteva N, Seethammagari M, et al. The E3 ligase c-Cbl regulates dendritic cell activation. EMBO Rep. 2011;12:971–9. 21799517 10.1038/embor.2011.143 PMC3166462

[26] Huang F, Gu H. Negative regulation of lymphocyte development and function by the Cbl family of proteins. Immunol Rev. 2008;224:229–38. 18759930 10.1111/j.1600-065X.2008.00655.x

[27] Liu YC, Gu H. Cbl and Cbl-b in T-cell regulation. Trends Immunol. 2002;23:140–3. 11864842 10.1016/s1471-4906(01)02157-3

[28] Tong H, Li X, Zhang J, Gong L, Sun W, Calderon V, et al. Ubiquitin Ligases CBL and CBL-B Maintain the homeostasis and immune quiescence of dendritic cells. Front Immunol. 2021;12:757231. 34630435 10.3389/fimmu.2021.757231 PMC8494778

[29] Xu F, Liu C, Dong Y, Wu W, Xu J, Yan Y, et al. Ablation of Cbl-b and c-Cbl in dendritic cells causes spontaneous liver cirrhosis via altering multiple properties of CD103(+) cDC1s. Cell Death Discov. 2022;8:142. 35354799 10.1038/s41420-022-00953-2 PMC8967913

[30] Yu W, Chen J, Xiong Y, Pixley FJ, Dai XM, Yeung YG, et al. CSF-1 receptor structure/function in MacCsf1r-/- macrophages: regulation of proliferation, differentiation, and morphology. J Leukoc Biol. 2008;84:852–63. 18519746 10.1189/jlb.0308171 PMC2516905

[31] Suzu S, Hiyoshi M, Yoshidomi Y, Harada H, Takeya M, Kimura F, et al. M-CSF-mediated macrophage differentiation but not proliferation is correlated with increased and prolonged ERK activation. J Cell Physiol. 2007;212:519–25. 17443671 10.1002/jcp.21045

[32] Eskandari E, Eaves CJ. Paradoxical roles of caspase-3 in regulating cell survival, proliferation, and tumorigenesis. J Cell Biol. 2022;221:e202201159. 10.1083/jcb.202201159 PMC9106709 35551578

[33] Nozaki K, Maltez VI, Rayamajhi M, Tubbs AL, Mitchell JE, Lacey CA, et al. Caspase-7 activates ASM to repair gasdermin and perforin pores. Nature. 2022;606:960–7. 35705808 10.1038/s41586-022-04825-8 PMC9247046

[34] Adlakha YK, Saini N. miR-128 exerts pro-apoptotic effect in a p53 transcription-dependent and -independent manner via PUMA-Bak axis. Cell Death Dis. 2013;4:e542. 23492773 10.1038/cddis.2013.46 PMC3613825

[35] Nakano K, Vousden KH. PUMA, a novel proapoptotic gene, is induced by p53. Mol Cell. 2001;7:683–94. 11463392 10.1016/s1097-2765(01)00214-3

[36] Wang P, Qiu W, Dudgeon C, Liu H, Huang C, Zambetti GP, et al. PUMA is directly activated by NF-kappaB and contributes to TNF-alpha-induced apoptosis. Cell Death Differ. 2009;16:1192–202. 19444283 10.1038/cdd.2009.51 PMC2872087

[37] Kim H, Rafiuddin-Shah M, Tu HC, Jeffers JR, Zambetti GP, Hsieh JJ, et al. Hierarchical regulation of mitochondrion-dependent apoptosis by BCL-2 subfamilies. Nat Cell Biol. 2006;8:1348–58. 17115033 10.1038/ncb1499

[38] Yu J, Zhang L, Hwang PM, Kinzler KW, Vogelstein B. PUMA induces the rapid apoptosis of colorectal cancer cells. Mol Cell. 2001;7:673–82. 11463391 10.1016/s1097-2765(01)00213-1

[39] Manne RK, Agrawal Y, Malonia SK, Banday S, Edachery S, Patel A, et al. FBXL20 promotes breast cancer malignancy by inhibiting apoptosis through degradation of PUMA and BAX. J Biol Chem. 2021;297:101253. 34587475 10.1016/j.jbc.2021.101253 PMC8507197

[40] Amacher JF, Hobbs HT, Cantor AC, Shah L, Rivero MJ, Mulchand SA, et al. Phosphorylation control of the ubiquitin ligase Cbl is conserved in choanoflagellates. Protein Sci Publ Protein Soc. 2018;27:923–32. 10.1002/pro.3397 PMC5916117 29498112

[41] Dou H, Buetow L, Hock A, Sibbet GJ, Vousden KH, Huang DT. Structural basis for autoinhibition and phosphorylation-dependent activation of c-Cbl. Nat Struct Mol Biol. 2012;19:184–92. 22266821 10.1038/nsmb.2231 PMC3880865

[42] Huang L, Thiex NW, Lou J, Ahmad G, An W, Low-Nam ST, et al. The ubiquitin ligases Cbl and Cbl-b regulate macrophage growth by controlling CSF-1R import into macropinosomes. Mol Biol Cell. 2024;35:ar38. 38170572 10.1091/mbc.E23-09-0345 PMC10916879

[43] Pyonteck SM, Akkari L, Schuhmacher AJ, Bowman RL, Sevenich L, Quail DF, et al. CSF-1R inhibition alters macrophage polarization and blocks glioma progression. Nat Med. 2013;19:1264–72. 24056773 10.1038/nm.3337 PMC3840724

[44] Klemm F, Möckl A, Salamero-Boix A, Alekseeva T, Schäffer A, Schulz M, et al. Compensatory CSF2-driven macrophage activation promotes adaptive resistance to CSF1R inhibition in breast-to-brain metastasis. Nat Cancer. 2021;2:1086–101. 35121879 10.1038/s43018-021-00254-0

[45] Bohannon DG, Zablocki-Thomas LD, Leung ES, Dupont JK, Hattler JB, Kowalewska J, et al. CSF1R inhibition depletes brain macrophages and reduces brain virus burden in SIV-infected macaques. Brain J Neurol. 2024;147:3059–69. 10.1093/brain/awae153 PMC11370798 39049445

[46] Audu CO, Melvin WJ, Joshi AD, Wolf SJ, Moon JY, Davis FM, et al. Macrophage-specific inhibition of the histone demethylase JMJD3 decreases STING and pathologic inflammation in diabetic wound repair. Cell Mol Immunol. 2022;19:1251–62. 36127466 10.1038/s41423-022-00919-5 PMC9622909

[47] Davis FM, Kimball A, denDekker A, Joshi AD, Boniakowski AE, Nysz D, et al. Histone Methylation Directs Myeloid TLR4 Expression and Regulates Wound Healing following Cutaneous Tissue Injury. J Immunol. 2019;202:1777–85. 30710046 10.4049/jimmunol.1801258 PMC6401313

[48] Menon MB, Yakovleva T, Ronkina N, Suwandi A, Odak I, Dhamija S, et al. Lyz2-Cre-Mediated Genetic Deletion of Septin7 Reveals a Role of Septins in Macrophage Cytokinesis and Kras-Driven Tumorigenesis. Front Cell Dev Biol. 2021;9:795798. 35071236 10.3389/fcell.2021.795798 PMC8772882

[49] Guilliams M, De Kleer I, Henri S, Post S, Vanhoutte L, De Prijck S, et al. Alveolar macrophages develop from fetal monocytes that differentiate into long-lived cells in the first week of life via GM-CSF. J Exp Med. 2013;210:1977–92. 24043763 10.1084/jem.20131199 PMC3782041

[50] Gschwend J, Sherman SPM, Ridder F, Feng X, Liang HE, Locksley RM, et al. Alveolar macrophages rely on GM-CSF from alveolar epithelial type 2 cells before and after birth. J Exp Med. 2021;218:e20210745. 10.1084/jem.20210745 PMC8404471 34431978

[51] Reed JA, Ikegami M, Robb L, Begley CG, Ross G, Whitsett JA. Distinct changes in pulmonary surfactant homeostasis in common beta-chain- and GM-CSF-deficient mice. Am J Physiol Lung Cell Mol Physiol. 2000;278:L1164–71. 10835321 10.1152/ajplung.2000.278.6.L1164

[52] Finley D. Recognition and processing of ubiquitin-protein conjugates by the proteasome. Annu Rev Biochem. 2009;78:477–513. 19489727 10.1146/annurev.biochem.78.081507.101607 PMC3431160

[53] Erpapazoglou Z, Walker O, Haguenauer-Tsapis R. Versatile roles of k63-linked ubiquitin chains in trafficking. Cells. 2014;3:1027–88. 25396681 10.3390/cells3041027 PMC4276913

[54] Al-Hakim A, Escribano-Diaz C, Landry MC, O’Donnell L, Panier S, Szilard RK, et al. The ubiquitous role of ubiquitin in the DNA damage response. DNA Repair. 2010;9:1229–40. 21056014 10.1016/j.dnarep.2010.09.011 PMC7105183

[55] Wang G, Gao Y, Li L, Jin G, Cai Z, Chao JI, et al. K63-linked ubiquitination in kinase activation and cancer. Front Oncol. 2012;2:5. 22649774 10.3389/fonc.2012.00005 PMC3355940

[56] Ordureau A, Münch C, Harper JW. Quantifying ubiquitin signaling. Mol Cell. 2015;58:660–76. 26000850 10.1016/j.molcel.2015.02.020 PMC4441763

[57] Hou P, Yang K, Jia P, Liu L, Lin Y, Li Z, et al. A novel selective autophagy receptor, CCDC50, delivers K63 polyubiquitination-activated RIG-I/MDA5 for degradation during viral infection. Cell Res. 2021;31:62–79. 32612200 10.1038/s41422-020-0362-1 PMC7852694

[58] Zhou Z, Zhu X, Yin R, Liu T, Yang S, Zhou L, et al. K63 ubiquitin chains target NLRP3 inflammasome for autophagic degradation in ox-LDL-stimulated THP-1 macrophages. Aging. 2020;12:1747–59. 32003754 10.18632/aging.102710 PMC7053591

[59] Zhou X, Zhao Y, Huang S, Shu H, Zhang Y, Yang H, et al. TRIM32 promotes neuronal ferroptosis by enhancing K63-linked ubiquitination and subsequent p62-selective autophagic degradation of GPX4. Int J Biol Sci. 2025;21:1259–74. 39897031 10.7150/ijbs.106690 PMC11781169

[60] Lee PS, Wang Y, Dominguez MG, Yeung YG, Murphy MA, Bowtell DD, et al. The Cbl protooncoprotein stimulates CSF-1 receptor multiubiquitination and endocytosis, and attenuates macrophage proliferation. EMBO J. 1999;18:3616–28. 10393178 10.1093/emboj/18.13.3616 PMC1171440

[61] Krönke J, Udeshi ND, Narla A, Grauman P, Hurst SN, McConkey M, et al. Lenalidomide causes selective degradation of IKZF1 and IKZF3 in multiple myeloma cells. Science. 2014;343:301–5. 24292625 10.1126/science.1244851 PMC4077049

[62] Tan X, Calderon-Villalobos LI, Sharon M, Zheng C, Robinson CV, Estelle M, et al. Mechanism of auxin perception by the TIR1 ubiquitin ligase. Nature. 2007;446:640–5. 17410169 10.1038/nature05731

[63] Huang HL, Weng HY, Wang LQ, Yu CH, Huang QJ, Zhao PP, et al. Triggering Fbw7-mediated proteasomal degradation of c-Myc by oridonin induces cell growth inhibition and apoptosis. Mol Cancer Ther. 2012;11:1155–65. 22389469 10.1158/1535-7163.MCT-12-0066

