# Supplementary material for: E3 Ubiquitin ligases Cbl-b and c-Cbl maintain the homeostasis of macrophages by regulating the M-CSF/M-CSFR signaling axis
Source: Cell Death Dis. 2025 Oct 7;16(1):716. doi: 10.1038/s41419-025-08047-4 (PMC12504418; doi:10.1038/s41419-025-08047-4)
Supplement: Supplementary file 1 — Supplemental Material [file 41419_2025_8047_MOESM1_ESM.docx]

**Supplementary information**

**
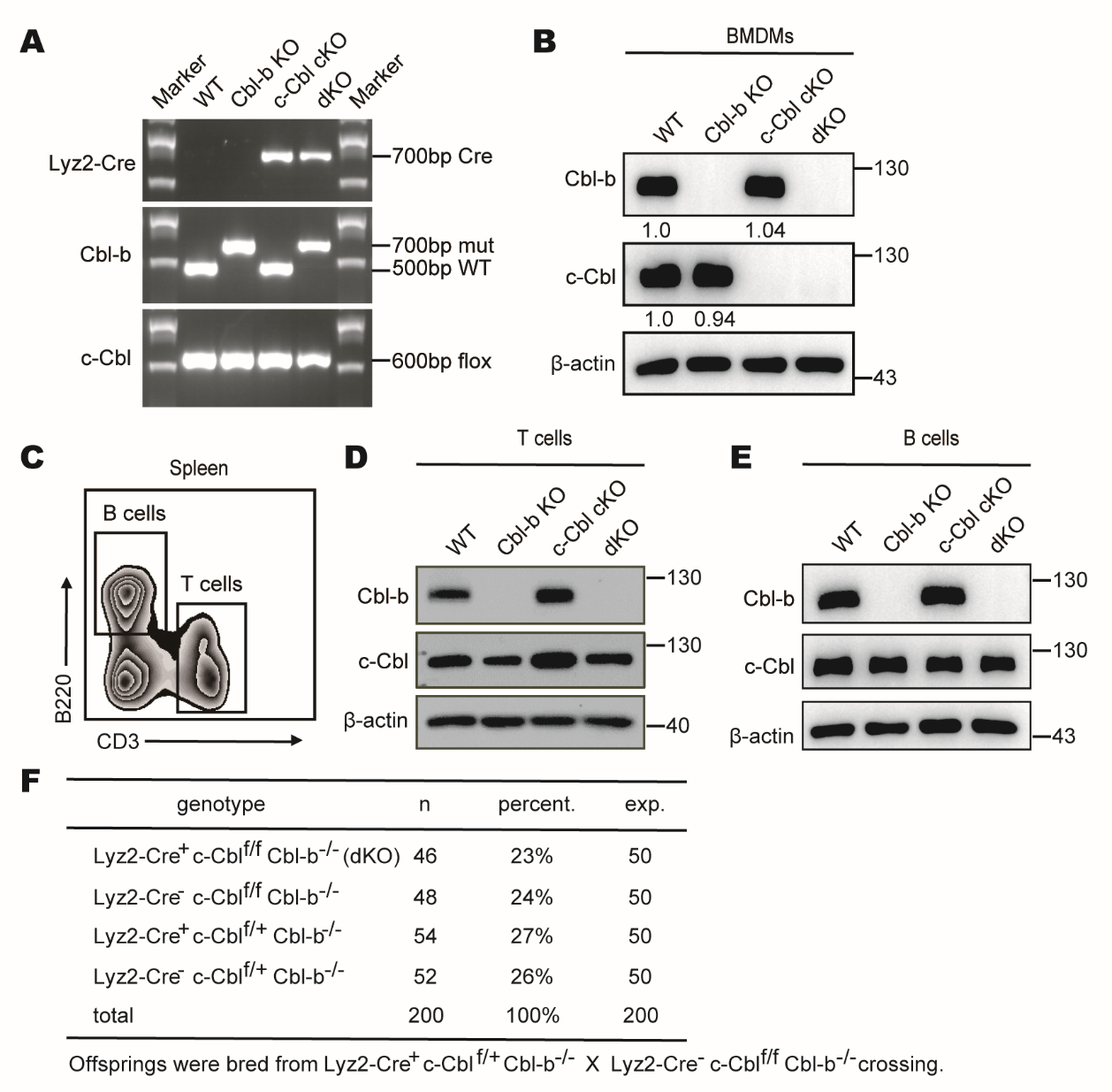
**

**Supplementary Figure. 1 Identification of dKO mice.**

(**A**) Genomic DNA was extracted and PCR was performed to detect levels of Lyz2, Cbl-b and c-Cbl in WT, Cbl-b KO, c-Cbl cKO and dKO mice. (**B**) Western blot analysis of Cbl-b and c-Cbl expression in WT, Cbl-b KO, c-Cbl cKO and dKO BMDMs. (**C**) FACS sorting strategy for purifying splenic T and B cells. (**D**) Western blot analysis of Cbl-b and c-Cbl expression in WT, Cbl-b KO, c-Cbl cKO and dKO T cells. (**E**) Western blot analysis of Cbl-b and c-Cbl expression in WT, Cbl-b KO, c-Cbl cKO and dKO B cells. (**F**) Genotypes of offspring from Lyz2-Cre^+^ c-Cbl^f/+^ Cbl-b^-/-^ X Lyz2-Cre^-^ c-Cbl^f/f^ Cbl-b^-/-^ crossings.

**
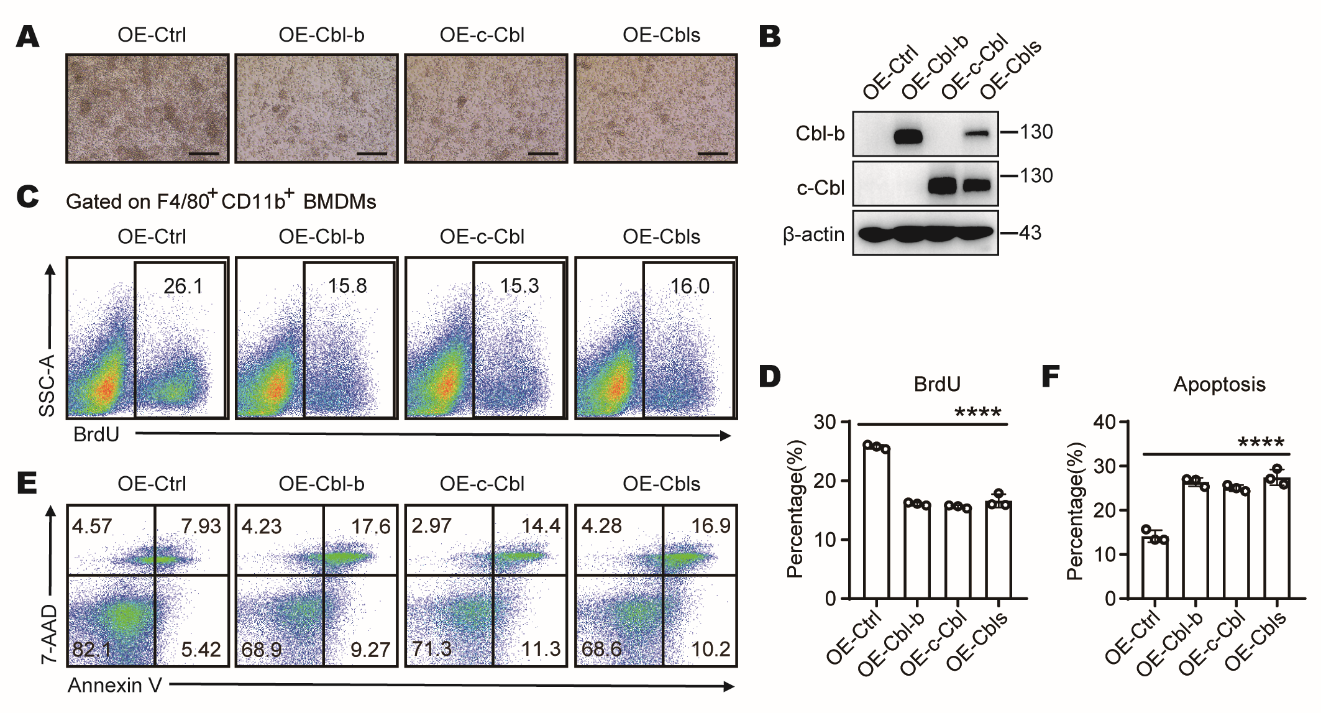
**

**Supplementary Figure. 2 Reestablish the expression of Cbls in dKO BMDMs.**

(**A**) Microscopy of clonal morphology of dKO (OE-Ctrl), dKO (OE-Cbl-b), dKO (OE-c-Cbl) and dKO (OE-Cbls) BMDMs generated in M-CSF dependent BM cell culture (r=3 per group); scale bar, 100 μm. (**B**) Bone marrow cells from dKO mice infected with OE-Ctrl, OE-Cbl-b, OE-c-Cbl, and OE-Cbls viruses while simultaneously inducing cell differentiation into BMDMs using M-CSF. And BMDMs were harvested for western blot analysis of Cbl-b and c-Cbl. (**C and D**) Bone marrow cells from dKO mice infected with OE-Ctrl, OE-Cbl-b, OE-c-Cbl, and OE-Cbls viruses while simultaneously inducing cell differentiation into BMDMs using M-CSF. And BMDMs were harvested for proliferation analysis. Shown are FACS analyses (**C**) and statistics (**D**) of BrdU^+^ F4/80^+^ CD11b^+^ cells (*r*=3 per group). (**E and F**) Bone marrow cells from dKO mice infected with OE-Ctrl, OE-Cbl-b, OE-c-Cbl, and OE-Cbls viruses while simultaneously inducing cell differentiation into BMDMs using M-CSF. And BMDMs were harvested for apoptosis analysis. Shown are FACS analyses (**E**) and statistics (**F**) of Annexin V^+^ 7AAD^−^ and Annexin V^+^ 7AAD^+^ F4/80^+^ CD11b^+^ cells (*r*=3 per group). The "*r*" represents the number of times the technology is repeated. One-Way ANOVA comparisons for **D and F.** *****p*<0.0001. *p*<0.05 was considered statistically significant.

**
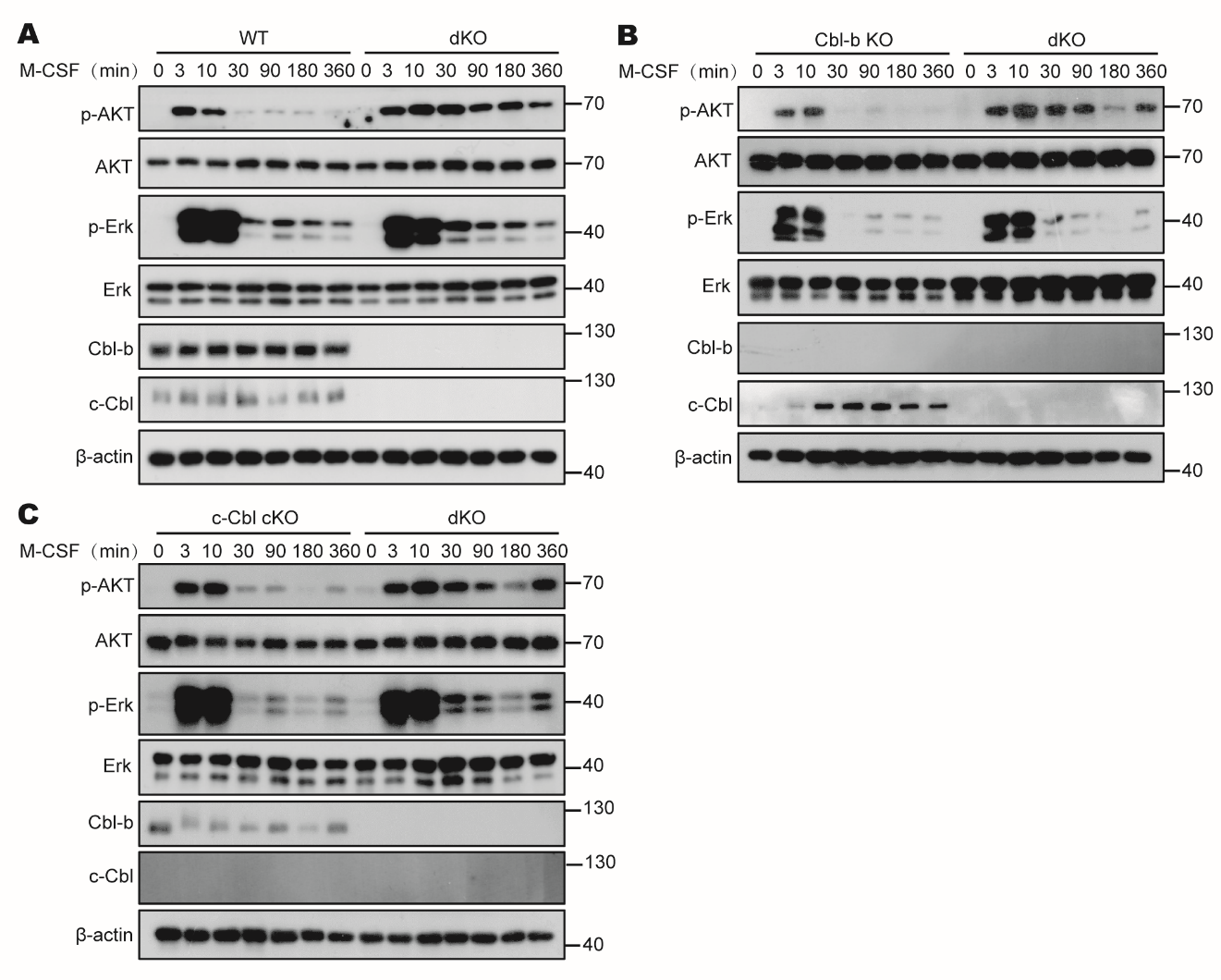
**

**Supplementary Figure. 3 dKO BMDMs exhibit prolonged AKT and Erk activation upon M-CSF stimulation.**

(**A**) WT and dKO BMDMs were starved of M-CSF for 24 h and restimulated with M-CSF (50 ng/mL) for indicated times and harvested for western blot analysis of p-AKT and p-Erk. (**B**) Cbl-b KO and dKO BMDMs were starved of M-CSF for 24 h and restimulated with M-CSF (50 ng/mL) for indicated times and harvested for western blot analysis of p-AKT and p-Erk. (**C**) c-Cbl cKO and dKO BMDMs were starved of M-CSF for 24 h and restimulated with M-CSF (50 ng/mL) for indicated times and harvested for western blot analysis of p-AKT and p-Erk.


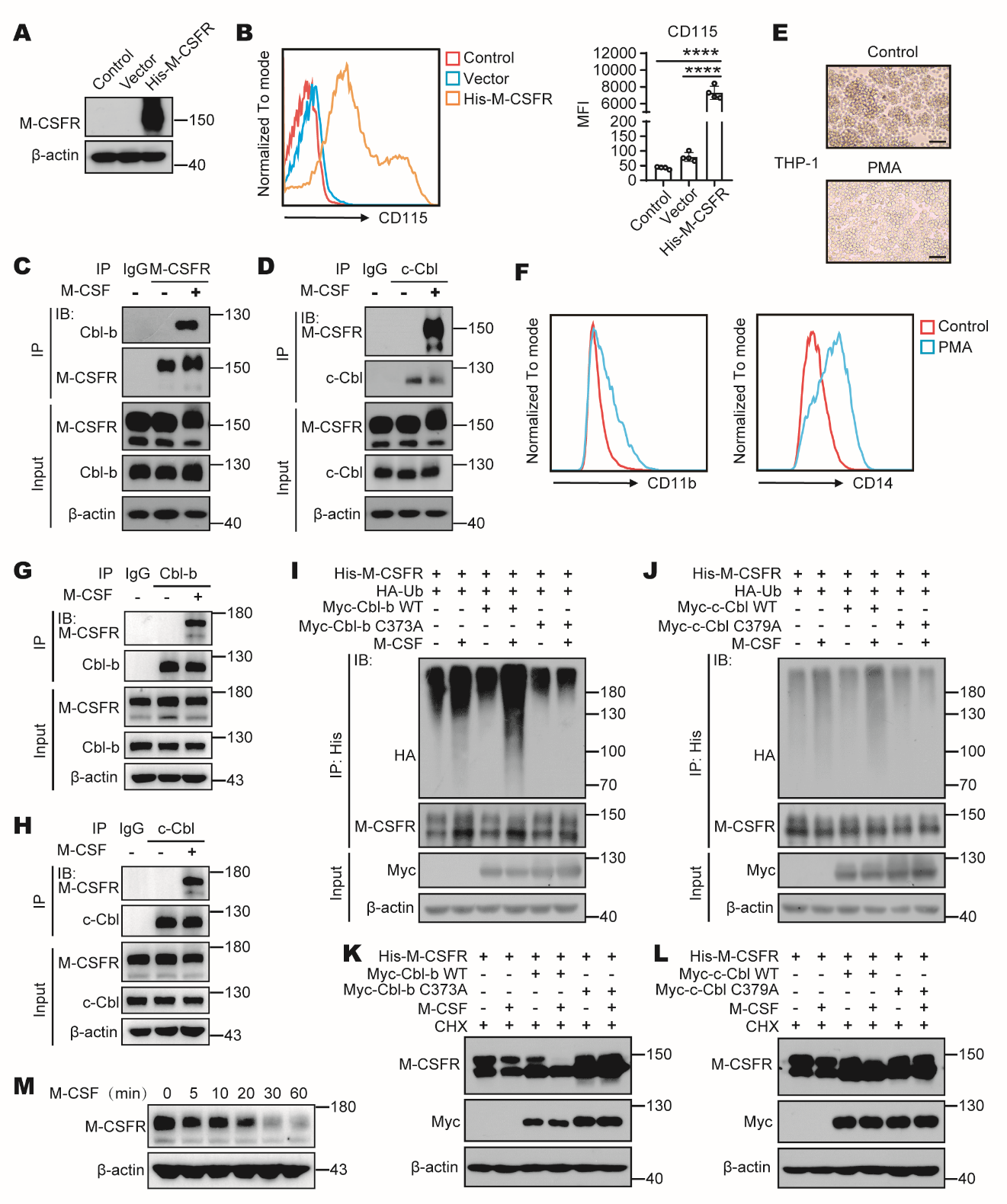


**Supplementary Figure. 4 Cbls mediate M-CSF-induced the ubiquitination and degradation of M-CSFR dependent on their ubiquitination enzyme activity.**

(**A**) Western blot analysis of M-CSFR expression in HEK293T cells transfected with His-M-CSFR. (**B**) Flow cytometry analysis of cell surface expression of CD115 (M-CSFR) in HEK293T cells transfected with His-M-CSFR (*r*=3 per group). (**C**) Immunoprecipitation analysis of endogenous interaction M-CSFR and Cbl-b in MH-S cells treated with M-CSF (50 ng/mL). (**D**) Immunoprecipitation analysis of endogenous interaction M-CSFR and c-Cbl in MH-S cells treated with M-CSF (50 ng/mL). (**E**) Morphological comparison of THP-1 cells before and after PMA (100 ng/mL) induction using light microscope (r=3 per group); scale bar, 100 μm. (**F**) Flow cytometry analysis of cell surface expression of CD11b and CD14 in THP-1 cells before and after PMA induction (r=3 per group). (**G**) Immunoprecipitation analysis of endogenous interaction M-CSFR and Cbl-b in PMA-stimulated THP-1 cells treated with M-CSF (50 ng/mL). (**H**) Immunoprecipitation analysis of endogenous interaction M-CSFR and c-Cbl in PMA-stimulated THP-1 cells treated with M-CSF (50 ng/mL). (**I**) Immunoprecipitation analysis of polyubiquitination of M-CSFR in HEK293T cells cotransfected with His-M-CSFR, HA-Ub and Myc-Cbl-b (WT or inactive C373A mutant) and then treated with M-CSF (50  ng/mL) for 2 h. (**J**) Immunoprecipitation analysis of polyubiquitination of M-CSFR in HEK293T cells cotransfected with His-M-CSFR, HA-Ub and Myc-c-Cbl (WT or inactive C379A mutant) and then treated with M-CSF (50  ng/mL) for 2 h. (**K**) Western blot analysis of M-CSFR in HEK293T cells cotransfected with His-M-CSFR and Myc-Cbl-b (WT or inactive C373A mutant) and then treated with M-CSF (50  ng/mL) and CHX (50 µM) for 2 h. (**L**) Western blot analysis of M-CSFR in HEK293T cells cotransfected with His-M-CSFR and Myc-c-Cbl (WT or inactive C379A mutant) and then treated with M-CSF (50  ng/mL) and CHX (50 µM) for 2 h. (**M**) PMA-stimulated THP-1 cells were treated with M-CSF (50 ng/mL) for indicated times and harvested for western blot analysis of degradation of M-CSFR. The "*r*" represents the number of times the technology is repeated. Unpaired Student’s *t* test for **B**. *****p*<0.0001. *p*<0.05 was considered statistically significant.


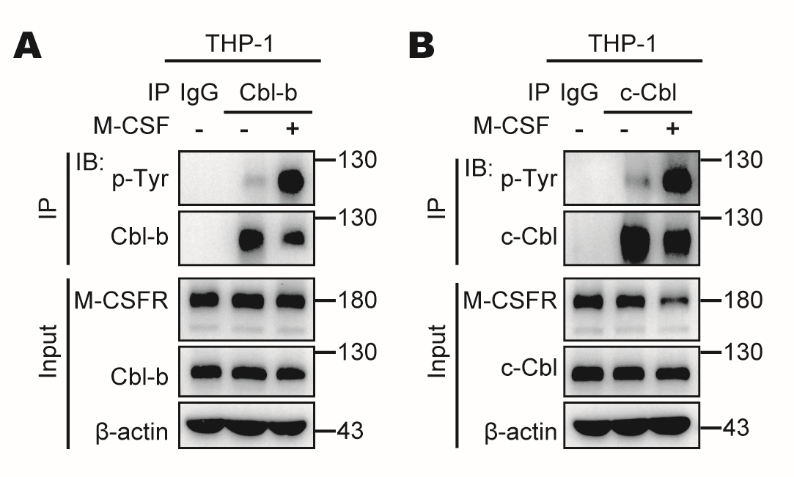


**Supplementary Figure. 5 M-CSF induced the tyrosine phosphorylation of Cbls in PMA-stimulated THP-1 cells.**

(**A**) Immunoprecipitation analysis of tyrosine phosphorylation of Cbl-b in PMA-stimulated THP-1 cells treated with M-CSF (50 ng/mL). (**B**) Immunoprecipitation analysis of tyrosine phosphorylation of c-Cbl in PMA-stimulated THP-1 cells treated with M-CSF (50 ng/mL).


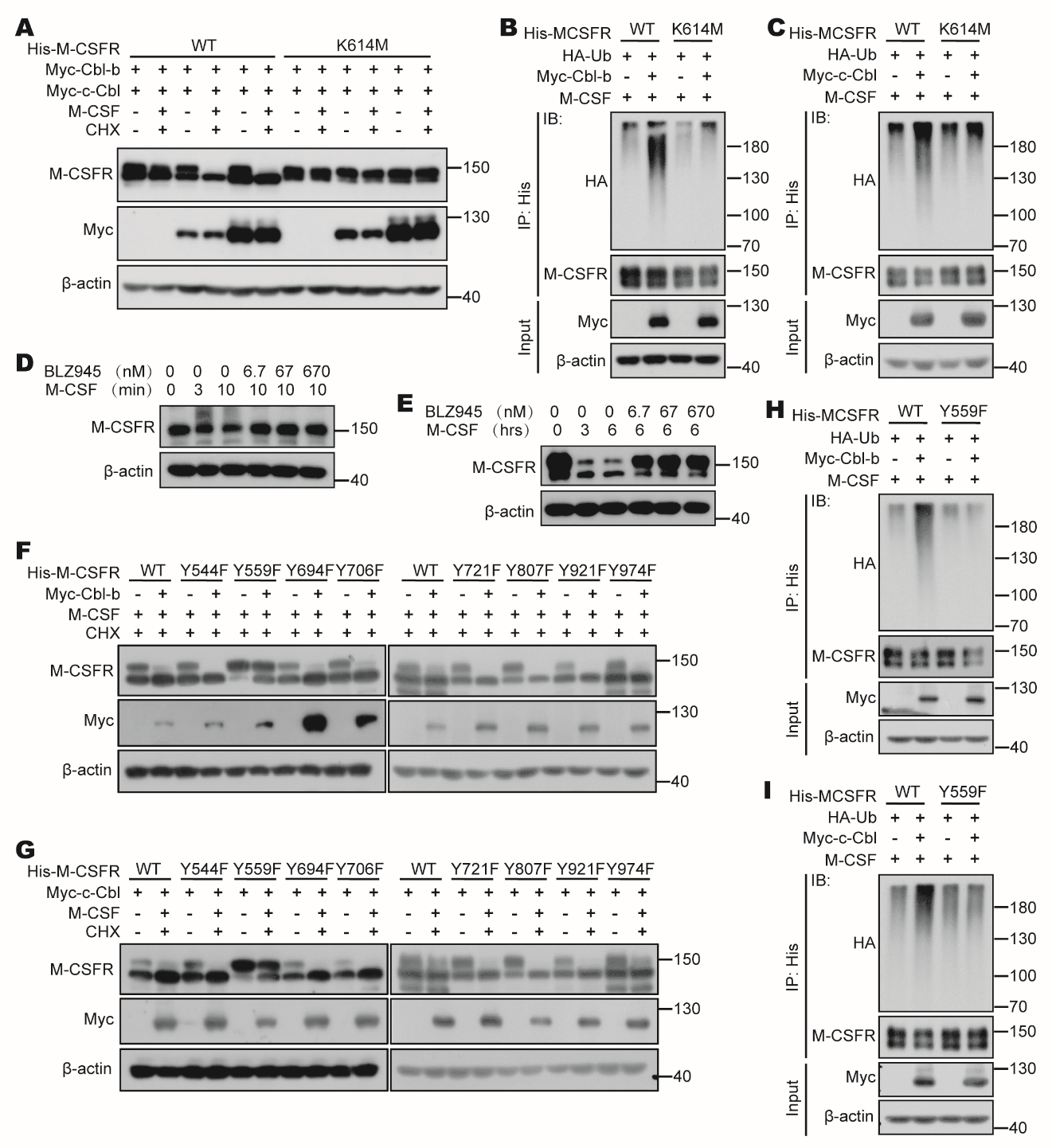


**Supplementary Figure. 6 Autophosphorylation of M-CSFR is critical for M-CSFR ubiquitination and degradation.**

(**A**) Western blot analysis of M-CSFR in HEK293T cells cotransfected with His-M-CSFR (WT or K614M) and Myc-Cbl (Cbl-b or c-Cbl) and then treated with M-CSF (50  ng/mL) and CHX (50 µM) for 2 h. (**B**) Immunoprecipitation analysis of polyubiquitination of M-CSFR in HEK293T cells cotransfected with His-M-CSFR (WT or K614M), HA-Ub and Myc-Cbl-b and then treated with M-CSF (50  ng/mL) for 2 h. (**C**) Immunoprecipitation analysis of polyubiquitination of M-CSFR in HEK293T cells cotransfected with His-M-CSFR (WT or K614M), HA-Ub and Myc-c-Cbl and then treated with M-CSF (50  ng/mL) for 2 h. (**D and E**) WT BMDMs were starved of M-CSF for 24 h and treated with M-CSF (50 ng/mL) and different concentrations of BLZ945 for indicated times and harvested for western blot analysis of M-CSFR. (**F**) Western blot analysis of M-CSFR in HEK293T cells cotransfected with His-M-CSFR (WT or its tyrosine mutants) and Myc-Cbl-b and then treated with M-CSF (50  ng/mL) and CHX (50 µM) for 2 h. (**G**) Western blot analysis of M-CSFR in HEK293T cells cotransfected with His-M-CSFR (WT or its tyrosine mutants) and Myc-c-Cbl and then treated with M-CSF (50  ng/mL) and CHX (50 µM) for 2 h. (**H**) Immunoprecipitation analysis of polyubiquitination of M-CSFR in HEK293T cells cotransfected with His-M-CSFR (WT or Y559F), HA-Ub and Myc-Cbl-b and then treated with M-CSF (50  ng/mL) for 2 h. (**I**) Immunoprecipitation analysis of polyubiquitination of M-CSFR in HEK293T cells cotransfected with His-M-CSFR (WT or Y559F), HA-Ub and Myc-c-Cbl and then treated with M-CSF (50  ng/mL) for 2 h.


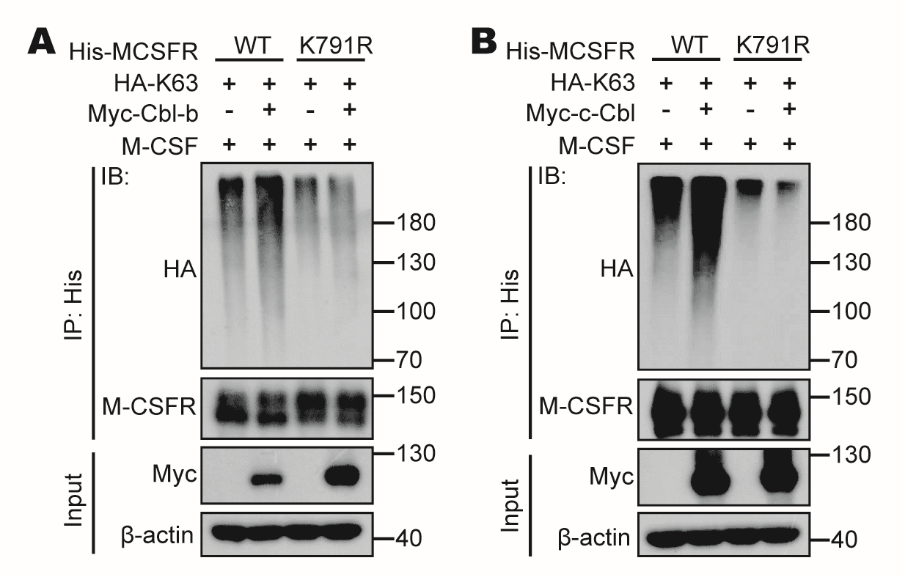


**Supplementary Figure. 7 Cbls mediate the K63-linked polyubiquitination modification at Lys791 of M-CSFR.**

(**A**) Immunoprecipitation analysis of K63-linked polyubiquitination of M-CSFR in HEK293T cells cotransfected with His-M-CSFR (WT or K791R), HA-K63 and Myc-Cbl-b and then treated with M-CSF (50  ng/mL) for 2 h. (**B**) Immunoprecipitation analysis of K63-linked polyubiquitination of M-CSFR in HEK293T cells cotransfected with His-M-CSFR (WT or K791R), HA-K63 and Myc-c-Cbl and then treated with M-CSF (50  ng/mL) for 2 h.


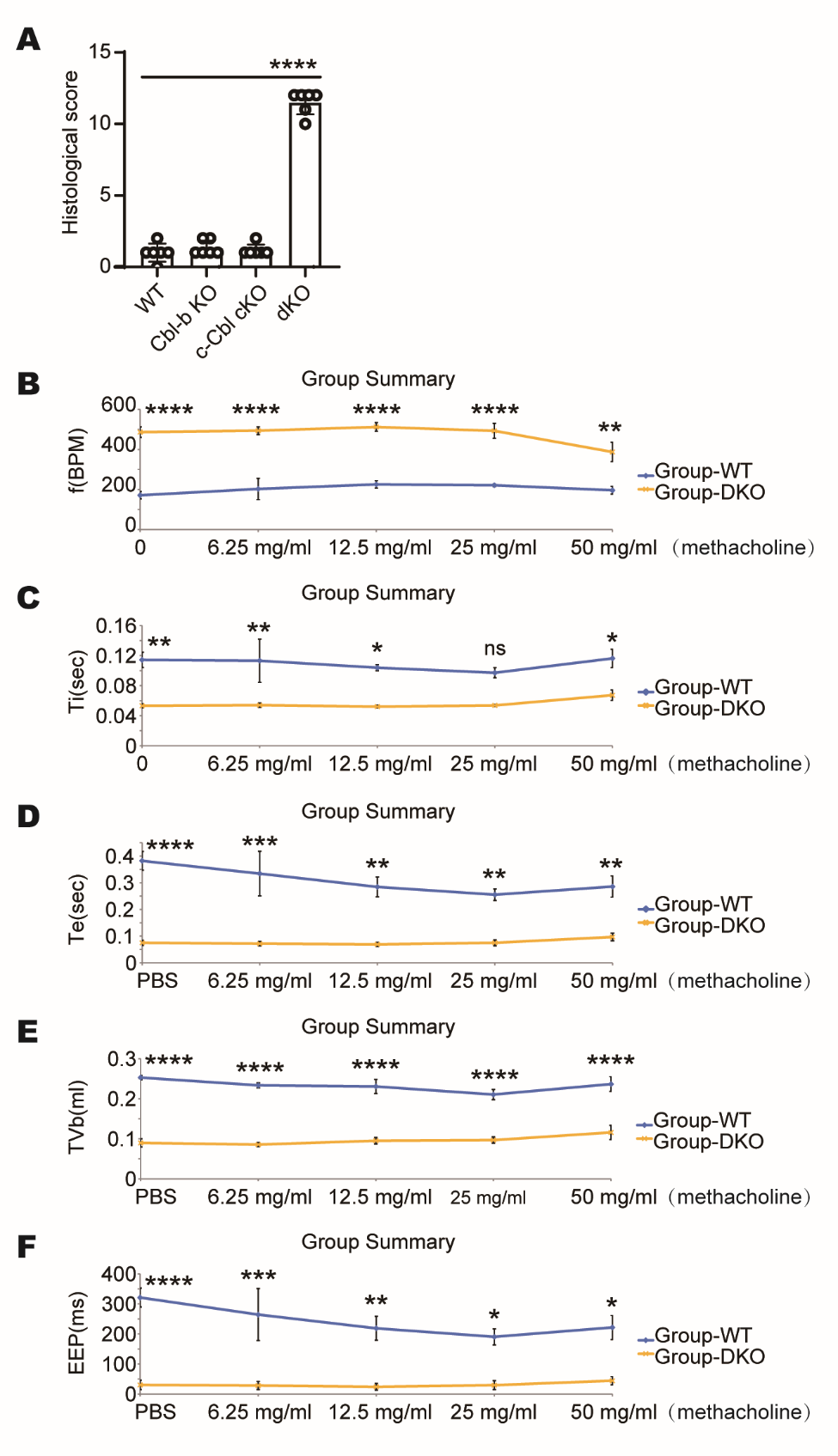


**Supplementary Figure. 8 Histopathological grading and pulmonary function tests of dKO mice.**

(**A**) Histopathological grading (multi-parameter comprehensive scoring method) of H&E staining sections of lung tissues in WT, Cbl-b KO, c-Cbl cKO and dKO mice (n=6 per group). (**B**) Pulmonary function assessment. The breathing frequence (f) was measured by the WBP system (n=6 per group). (**C**) Pulmonary function assessment. The inspiratory time (Ti) was measured by the WBP system (n=6 per group). (**D**) Pulmonary function assessment. The expiratory time (Te) was measured by the WBP system (n=6 per group). (**E**) Pulmonary function assessment. The tidal volume, baseline (TVb) was measured by the WBP system (n=6 per group). (**F**) Pulmonary function assessment. The expiratory end pressure (EEP) was measured by the WBP system (n=6 per group). The “*n*” represents the number of biologically independent samples. One-Way ANOVA comparisons for **A**, Two-Way ANOVA comparisons for B, C, D, E and F. *ns*, no significance, **p*<0.05, ***p*<0.01, ****p*<0.001, *****p*<0.0001. *p*<0.05 was considered statistically significant.


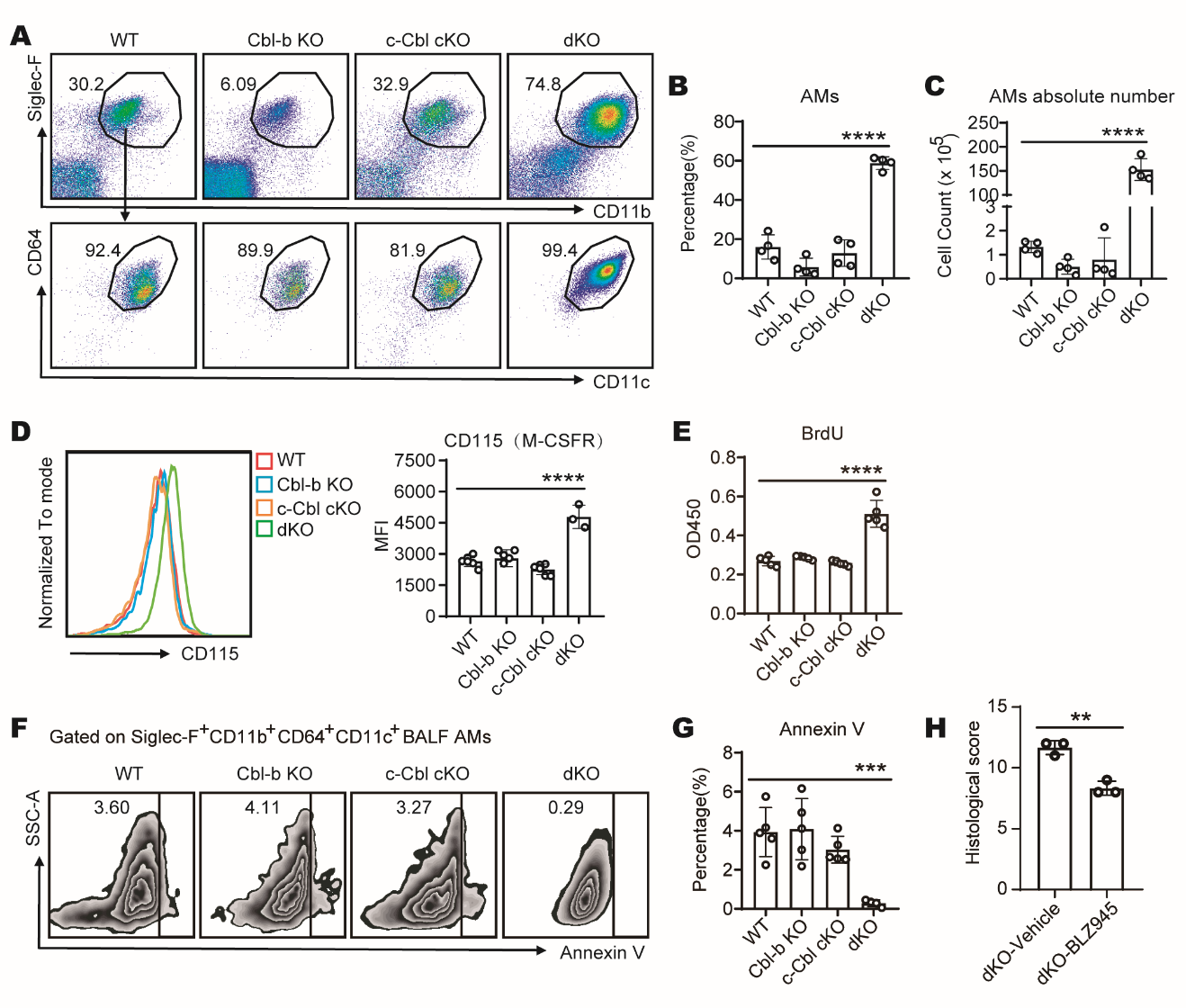


**Supplementary Figure. 9 The deficiency of Cbls contributes to the accumulation of macrophages in vivo.**

(**A**) Flow cytometry analysis of alveolar macrophages (AMs) (Siglec-F^+^ CD11b^+^ CD64^+^ CD11c^+^) in BALF from four groups of mice (n=4 per group). (**B and C**) Statistics of percentage (**B**) and absolute number (**C**) of AMs in BALF from four groups of mice (n=4 per group), as shown in A. (**D**) Flow cytometry analysis of cell surface expression of CD115 (M-CSFR) in WT, Cbl-b KO, c-Cbl cKO and dKO BALF AMs (n=3-5 per group). (**E**) Mice were injected with 1 mg BrdU 24 h and 6 h prior to sacrifice, respectively. Proliferation of AMs in BALF from four groups of mice was quantified using BrdU Cell Proliferation Assay Kit (n=5 per group). (**F**) Flow cytometry analysis of Annexin V^+^ AMs in BALF from four groups of mice (n=4 per group). (**G**) Statistics of percentage of Annexin V^+^ AMs in BALF from four groups of mice (n=4 per group), as shown in B. (**H**) Histopathological grading (multi-parameter comprehensive scoring method) of H&E staining sections of lung tissues in dKO mice (receiving treatment with either vehicle or BLZ945) (n=3 per group). The “*n*” represents the number of biologically independent samples. One-Way ANOVA comparisons for **B**, **C, D, E** and **G**. Unpaired Student’s *t* test for **H**. ***p*<0.01, ****p*<0.001, *****p*<0.0001. *p*<0.05 was considered statistically significant.


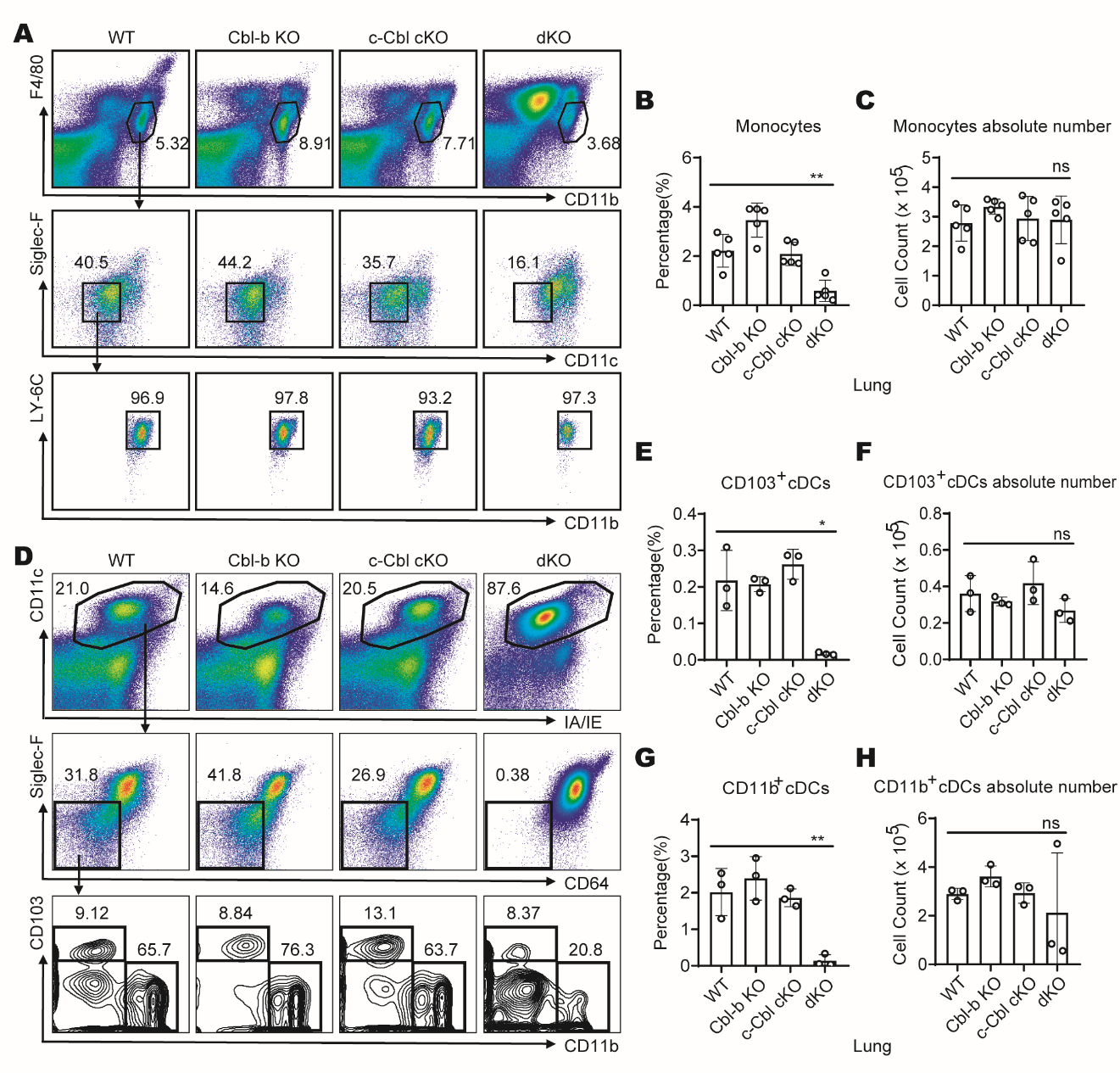


**Supplementary Figure. 10 There were no significant changes in monocytes and cDCs in dKO mice lungs.**

(**A**) Flow cytometry analysis of monocytes (F4/80^int^ CD11b^high^ Siglec-F^low^ CD11c^low^ LY-6C^high^) in lungs from four groups of mice (n=5 per group). (**B and C**) Statistics of percentage (**B**) and absolute number (**C**) of monocytes in lungs from four groups of mice (n=5 per group), as shown in A. (**D**) Flow cytometry analysis of CD103^+^ cDCs (CD11c^+^ IA/IE^+^ Siglec-F^-^ CD64^-^ CD103^+^) and CD11b^+^ cDCs (CD11c^+^ IA/IE^+^ Siglec-F^-^ CD64^-^ CD11b^+^) in lungs from four groups of mice (n=3 per group). (**E and F**) Statistics of percentage (**E**) and absolute number (**F**) of CD103^+^ cDCs in lungs from four groups of mice (n=3 per group), as shown in D. (**G and H**) Statistics of percentage (**G**) and absolute number (**H**) of CD11b^+^ cDCs in lungs from four groups of mice (n=3 per group), as shown in D. The “*n*” represents the number of biologically independent samples. One-Way ANOVA comparisons for **B**, **C, E, F, G** and **H**. *ns*, no significance, **p*<0.05, ***p*<0.01. *p*<0.05 was considered statistically significant.

**
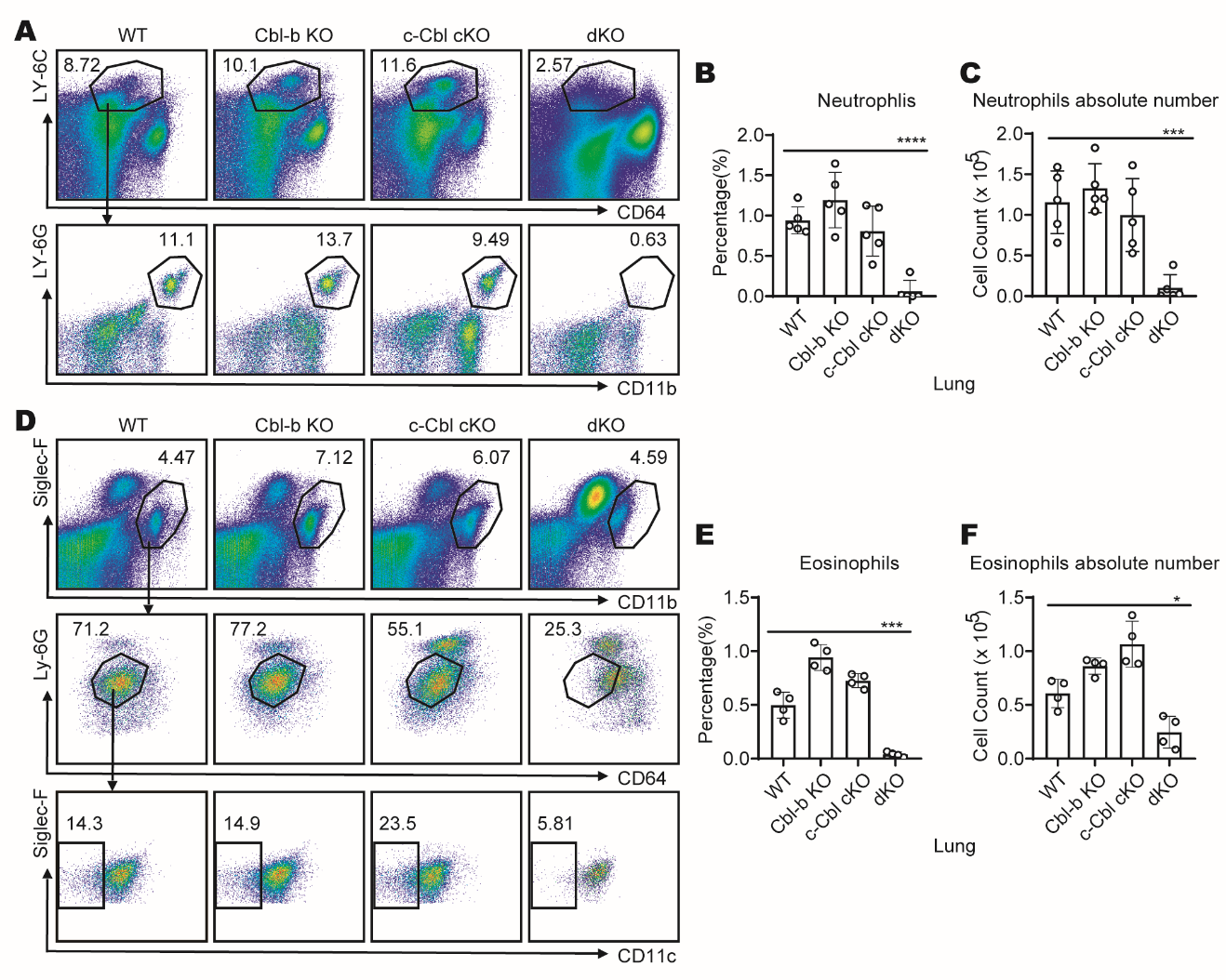
**

**Supplementary Figure. 11 Neutrophils and eosinophils were reduced in dKO mice lungs.**

(**A**) Flow cytometry analysis of neutrophlis (LY-6C^high^ CD64^low^ LY-6G^high^ CD11b^high^) in lungs from four groups of mice (n=5 per group). (**B and C**) Statistics of percentage (**B**) and absolute number (**C**) of neutrophlis in lungs from four groups of mice (n=5 per group), as shown in A. (**D**) Flow cytometry analysis of eosinophils (Siglec-F^high^ CD11b^high^ CD64^low^ LY-6C^int^ CD11c^-^) in lungs from four groups of mice (n=4 per group). (**E and F**) Statistics of percentage (**E**) and absolute number (**F**) of eosinophils in lungs from four groups of mice (n=4 per group), as shown in D. The “*n*” represents the number of biologically independent samples. One-Way ANOVA comparisons for **B**, **C, E and F**. **p*<0.05, ****p*<0.001, *****p*<0.0001. *p*<0.05 was considered statistically significant.


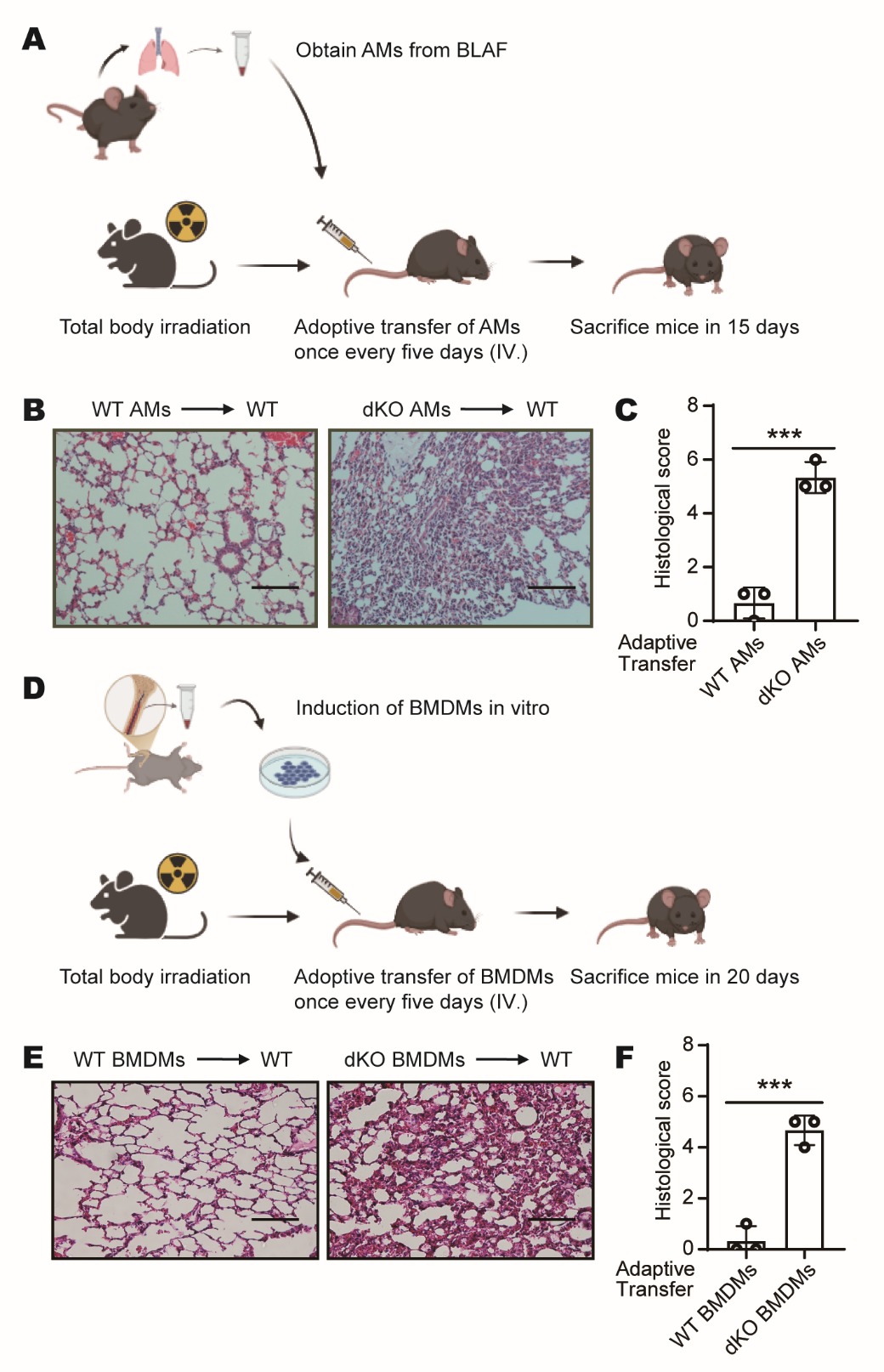


**Supplementary Figure. 12 DKO macrophages contribute to lung disease and injury.**

(**A and B**) BALF AMs purified from WT or dKO mice were adaptively transferred into WT recipient mice respectively, and lung sections were stained with H&E (n=3 per group); scale bar, 100 μm. (**C**) Histopathological grading (multi-parameter comprehensive scoring method) of H&E staining sections of lung tissues in WT recipient mice (adaptive transfer of WT or dKO AMs) (n=3 per group). (**D and E**) BMDMs induced from WT or dKO mice bone marrow were adaptively transferred into WT recipient mice respectively, and lung sections were stained with H&E (n=3 per group); scale bar, 100 μm. (**F**) Histopathological grading (multi-parameter comprehensive scoring method) of H&E staining sections of lung tissues in WT recipient mice (adaptive transfer of WT or dKO BMDMs) (n=3 per group). The “*n*” represents the number of biologically independent samples. Unpaired Student’s *t* test for **C** and **F**. *****p*<0.0001. *p*<0.05 was considered statistically significant.


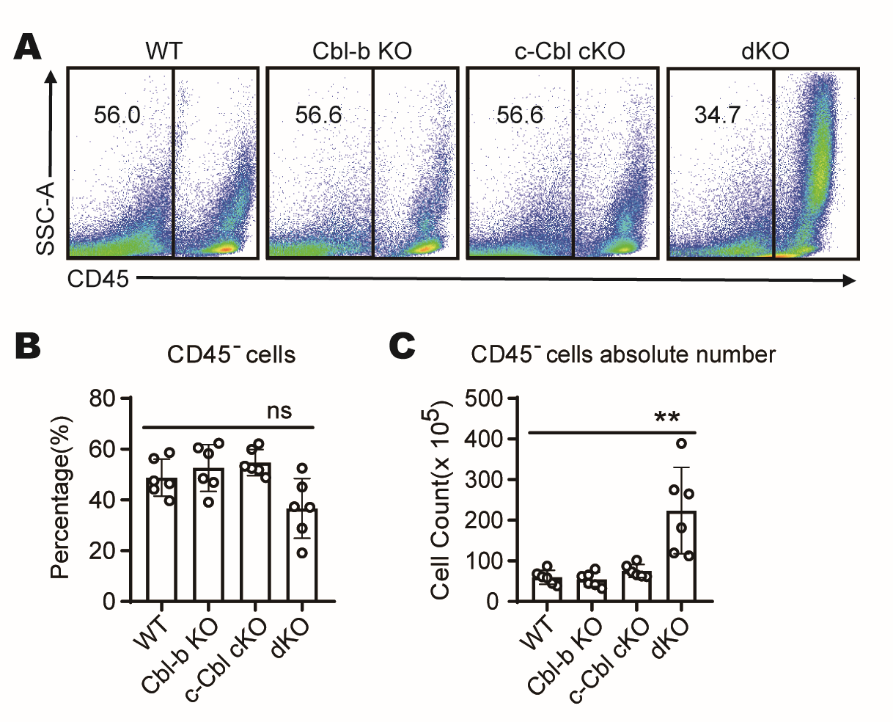


**Supplementary Figure. 13 CD45^-^ cells were increased in dKO mice lungs.**

(**A**) Flow cytometry analysis of CD45^-^ cells in lungs from four groups of mice (n=6 per group). (**B and C**) Statistics of percentage (**B**) and absolute number (**C**) of CD45^-^ cells in lungs from four groups of mice (n=6 per group), as shown in A. The “*n*” represents the number of biologically independent samples. One-Way ANOVA comparisons for **B** and **C**. *ns*, no significance, ***p*<0.01. *p*<0.05 was considered statistically significant.

**Supplementary Table 1.** **Primers for qPCR.**

| **Gene** |  | **Direction** | **Sequence 5’- to - 3’** |
| --- | --- | --- | --- |
| c-Myc |  | Forward | AATCCTGTACCTCGTCCGAT |
|  |  | Reverse | TCTTCTCCACAGACACCACA |
| Cyclin D1 |  | Forward | TGCTACCGCACAACGCA |
|  |  | Reverse | TCAATCTGTTCCTGGCAGGC |
| Cyclin D2 |  | Forward | CGTGTGATGCCCTGACTGAG |
|  |  | Reverse | GACTTGGATCCGGCGTTATG |
| M-CSFR |  | Forward | GCGATGTGTGAGCAATGGCA |
|  |  | Reverse | CGGATAATCGAACCTCGCCA |
| 18S |  | Forward | CGGCTACCACATCCAAGGAA |
|  |  | Reverse | GCTGGAATTACCGCGGCT |

**Supplementary Table 2. Primers for plasmid constructs.**

| **Gene** | **Direction** | **Sequence 5’- to - 3’** |
| --- | --- | --- |
| Cbl-b (pcDNA3.1+) | Forward | CGGGGTACCGCCACCATGGCAAATTCTATGAATGGCA |
|  | Reverse | CCGCTCGAGCTACAGATCCTCTTCTGAGATGAGTTTTTGTTCTAGATTCAGACGTGGGGAGA |
| c-Cbl (pcDNA3.1+) | Forward | CGGGGTACCGCCACCATGGCCGGCAACGTGAAGAA |
|  | Reverse | CCGCTCGAGCTACAGATCCTCTTCTGAGATGAGTTTTGTTCGGTGGCTACGTGAGCAGGA |
| M-CSFR (pcDNA3.1-) | Forward | CGGAATTCGCCACCATGGAGTTGGGGCCTCCTCT |
|  | Reverse | CCGCTCGAGTCAATGGTGATGGTGATGATGGCAGAACTGGTAGTTGTTAGG |
| Cbl-b (pMSGV) | Forward | TGGACCATCCTCTAGCCCTCGAGATGGCAAATTCTATGAATGGCAGA |
|  | Reverse | TTCCGGCTAGCCCTGCGCAAGCTTATTAGATTCAGACGTGGGGAGACA |
| c-Cbl (pMSGV) | Forward | TGGACCATCCTCTAGCCCTCGAGATGGCCGGCAACGTGAAGAAG |
|  | Reverse | TTCCGGCTAGCCCTGCGCAAGCTTATGGTGGCTACGTGAGCAGGAG |

**Supplementary Table 3. Primers for mutant Cbl-b and c-Cbl constructs.**

| **Gene** | **Direction** | **Sequence 5’- to - 3’** |
| --- | --- | --- |
| Cbl-b C373A | Forward | TTCTCTGCACAGATCTTGGCGAGCTGAAAAGTGGAGCC |
|  | Reverse | GGCTCCACTTTTCAGCTCGCCAAGATCTGTGCAGAGAA |
| Cbl-b Y363F | Forward | TGGAGCCCATTTCACAAAACAGTTCATACTGCTCC |
|  | Reverse | GGAGCAGTATGAACTGTTTTGTGAAATGGGCTCCA |
| Cbl-b Y664F | Forward | GAGGAGGGACATCGAATTCTTCAGTGGCAAGGTG |
|  | Reverse | CACCTTGCCACTGAAGAATTCGATGTCCCTCCTC |
| Cbl-b Y708F | Forward | GATGAAGGAATCTTGAATTCATCGTCATCATCTTCTACCGTG |
|  | Reverse | CACGGTAGAAGATGATGACGATGAATTCAAGATTCCTTCATC |
| Cbl-b Y889F | Forward | GGAGCTGGTCAAAGTCCTGCGAGGCTCT |
|  | Reverse | AGAGCCTCGCAGGACTTTGACCAGCTCC |
| c-Cbl C379A | Forward | CATTCTCAGCACATATCTTAGCCAGTTGAAATGTGGAGCCCA |
|  | Reverse | TGGGCTCCACATTTCAACTGGCTAAGATATGTGCTGAGAATG |
| c-Cbl Y369F | Forward | GAGCCCATTTCACAGAATAATTCATATTGTTCCTGGGTTACTTT |
|  | Reverse | AAAGTAACCCAGGAACAATATGAATTATTCTGTGAAATGGGCTC |
| c-Cbl Y672F | Forward | CAGCCAGAGAGAAAATGGCGTTGGCAGACG |
|  | Reverse | CGTCTGCCAACGCCATTTTCTCTCTGGCTG |
| c-Cbl Y698F | Forward | TGTGGGAGTCATAAATTCTGTGTCCTCTTCACTTTCC |
|  | Reverse | GGAAAGTGAAGAGGACACAGAATTTATGACTCCCACA |
| c-Cbl Y737F | Forward | TACATCGCTTCAAAGGTACAGCTGTCGATCTGCTG |
|  | Reverse | CAGCAGATCGACAGCTGTACCTTTGAAGCGATGTA |
| c-Cbl Y780F | Forward | GGCTTAGGCACATCAAAGCCATCATCCTCGTTTT |
|  | Reverse | AAAACGAGGATGATGGCTTTGATGTGCCTAAGCC |

**Supplementary Table 4. Primers for mutant M-CSFR constructs.**

| **Gene** | **Direction** | **Sequence 5’- to - 3’** |
| --- | --- | --- |
| M-CSFR K614M | Forward | CGTGGACTTTAGCATCATCACAGCCACCTTCAG |
|  | Reverse | CTGAAGGTGGCTGTGATGATGCTAAAGTCCACG |
| M-CSFR Y544F | Forward | GCGCACCTGGAACTTCGGCTTCTGCTTGTA |
|  | Reverse | TACAAGCAGAAGCCGAAGTTCCAGGTGCGC |
| M-CSFR Y559F | Forward | GTAGGGTCAATGAAGGTGAAGCTATTGCCTTCGTATC |
|  | Reverse | GATACGAAGGCAATAGCTTCACCTTCATTGACCCTAC |
| M-CSFR Y697F | Forward | CAGGTGGATGTTCTTGAAGCTGGAGTCTCCCTC |
|  | Reverse | GAGGGAGACTCCAGCTTCAAGAACATCCACCTG |
| M-CSFR Y706F | Forward | CCCTGCGCACAAATTTCTTCTCCAGGTGGATGT |
|  | Reverse | ACATCCACCTGGAGAAGAAATTTGTGCGCAGGG |
| M-CSFR Y721F | Forward | GCCTCATCTCCACGAAGGTGTCTACACCCTG |
|  | Reverse | CAGGGTGTAGACACCTTCGTGGAGATGAGGC |
| M-CSFR Y807F | Forward | CCCTTGACAACAAAGTTGGAGTCATTCATGATGTCC |
|  | Reverse | GGACATCATGAATGACTCCAACTTTGTTGTCAAGGG |
| M-CSFR Y921F | Forward | GCAGGTTAGCAAAGTCCTGGTCTCTCCTCTC |
|  | Reverse | GAGAGGAGAGACCAGGACTTTGCTAACCTGC |
| M-CSFR Y974F | Forward | GATGGCAGAACTGGAAGTTGTTAGGCTGCAGCAG |
|  | Reverse | CTGCTGCAGCCTAACAACTTCCAGTTCTGCCATC |
| M-CSFR K572R | Forward | CGAGGGAACTCCCACCTCTCA TTGTAGGGCA |
|  | Reverse | TGCCCTACAATGAGAGGTGGG AGTTCCCTCG |
| M-CSFR K584R | Forward | CCGGCTCCTAGAGTCCTACCA AACTGCAGGTT |
|  | Reverse | AACCTGCAGTTTGGTAGGACT CTAGGAGCCGG |
| M-CSFR K604R | Forward | GCACTGCATCTTCTCTGCCCA GACCAAAGGCT |
|  | Reverse | AGCCTTTGGTCTGGGCAGAGA AGATGCAGTGC |
| M-CSFR K698R | Forward | CTCCAGGTGGATGTTCCTGTA GCTGGAGTCTCC |
|  | Reverse | GGAGACTCCAGCTACAGGAA CATCCACCTGGAG |
| M-CSFR K791R | Forward | CCAAAGTCCCCAATCCTGGCC ACATGTCCGC |
|  | Reverse | GCGGACATGTGGCCAGGATT GGGGACTTTGG |
| M-CSFR K868R | Forward | TTTGGTATCCATCCCTCACCAGTTTGTAGAACTTGTTGTTC |
|  | Reverse | GAACAACAAGTTCTACAAACTGGTGAGGGATGGATACCAAA |
